# T3 and glucose increase expression of phosphoenolpyruvate carboxykinase (PCK1) leading to increased β-cell proliferation

**DOI:** 10.1016/j.molmet.2022.101646

**Published:** 2022-11-29

**Authors:** Liora S. Katz, Carmen Argmann, Luca Lambertini, Donald K. Scott

**Affiliations:** 1 Diabetes, Obesity and Metabolism Institute, Icahn School of Medicine at Mount Sinai, New York, NY, USA; 2 Department of Genetics and Genomics Sciences, Icahn School of Medicine at Mount Sinai, New York, NY, USA

**Keywords:** ChREBP, Diabetes, Pancreatic β-cell, Glucose, Thyroid hormone, Proliferation

## Abstract

**Objectives:**

Thyroid hormone (T3) and high glucose concentrations are critical components of β-cell maturation and function. In the present study, we asked whether T3 and glucose signaling pathways coordinately regulate transcription of genes important for β-cell function and proliferation.

**Methods:**

RNA-seq analysis was performed on cadaveric human islets from five different donors in response to low and high glucose concentrations and in the presence or absence of T3. Gene expression was also studies in sorted human β-cells, mouse islets and Ins-1 cells by RT-qPCR. Silencing of the thyroid hormone receptors (THR) was conducted using lentiviruses. Proliferation was assessed by ki67 immunostaining in primary human/mouse islets. Chromatin immunoprecipitation and proximity ligation assay were preformed to validate interactions of ChREBP and THR.

**Results:**

We found glucose-mediated expression of carbohydrate response element binding protein alpha and beta (ChREBPα and ChREBPβ) mRNAs and their target genes are highly dependent on T3 concentrations in rodent and human β-cells. In β-cells, T3 and glucose coordinately regulate the expression of ChREBPβ and PCK1 (phosphoenolpyruvate carboxykinase-1) among other important genes for β-cell maturation. Additionally, we show the thyroid hormone receptor (THR) and ChREBP interact, and their relative response elements are located near to each other on mutually responsive genes. In FACS-sorted adult human β-cells, we found that high concentrations of glucose and T3 induced the expression of PCK1. Next, we show that overexpression of Pck1 together with dimethyl malate (DMM), a substrate precursor, significantly increased β-cell proliferation in human islets. Finally, using a Cre-Lox approach, we demonstrated that ChREBPβ contributes to Pck1-dependent β-cell proliferation in mouse β-cells.

**Conclusions:**

We conclude that T3 and glucose act together to regulate ChREBPβ, leading to increased expression and activity of Pck1, and ultimately increased β-cell proliferation.

## Highlights

• T3 is necessary for and glucose-mediated transcription on rodent and human β-cells.
• T3 and glucose coordinately regulate the expression of ChREBPβ and PCK1 among other important genes for β-cell maturation.
• Fine-tuning of glucose and T3 signals can regulate gene expression and proliferation of rodent and human pancreatic β-cells.
• THR and ChREBP are interacting have common target genes and interact in close proximity in β-cells.
• PCK1 activity is sufficient to drive β-cell proliferation.

## Graphical abstract

**Figure:**
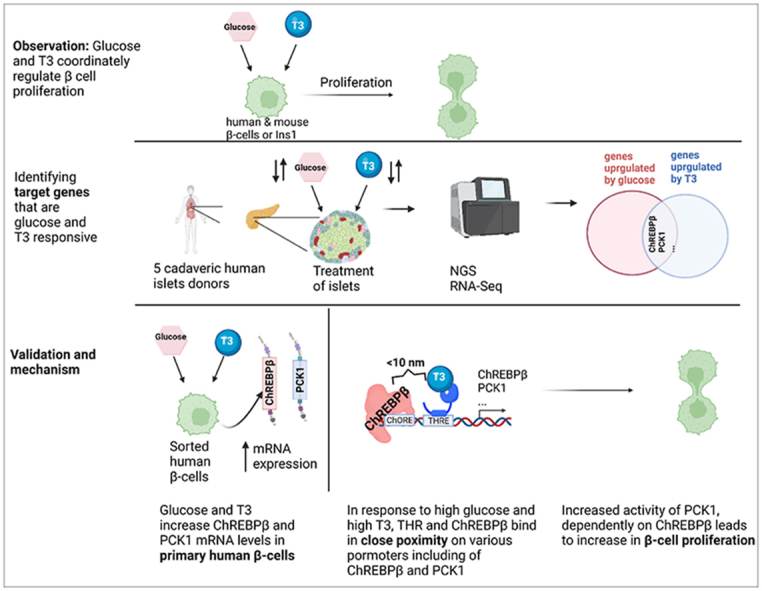
Created with BioRender.com

## 1 Introduction

The association between thyroid dysfunction and diabetes has long been recognized, and both hypothyroidism and hyperthyroidism are associated with diabetes [[1], [2], [3], [4], [5], [6], [7], [8], [9], [10]]. Thyroid hormones act to promote or antagonize insulin's actions depending on the context as well as the cell type they are acting upon. Thus, thyroid hormones participate in a fine balance that promotes normal glucose metabolism and any deviation of thyroid hormone abundance can perturb glucose homeostasis [4].

One way that T3 affects glucose homeostasis is through its influence on β-cell mass. Thyroid hormone (T3) is required for islet development and function [[11], [12], [13], [14], [15]]. T3 promotes β-cell proliferation in human and rodent cell lines and in the embryonic murine pancreas in explant culture [13,[16], [17], [18]]. Glucose is also a known β-cell mitogen, implicated in adaptive β-cell expansion [[19], [20], [21], [22]]. One transcription factor known to mediate this effect is Carbohydrate Response Element Binding Protein (ChREBP) [23,24]. ChREBP is a glucose responsive transcription factor that has two splice isoforms. One is ChREBPα which is mostly cytoplasmic and repressed in low glucose. The protein consists of an N-terminal low glucose inhibitory domain, containing a nuclear export signal that folds over and represses the activation domain. The C-terminal contains a beta-helix-loop-helix Zip DNA-binding domain. The other major isoform is ChREBPβ, which is a product of alternative splicing Where the low glucose inhibitory domain and nuclear export signals are removed but is otherwise identical to ChREPBα [25]. Consequently, ChREBPβ is mostly nuclear, and is constitutively and potently active [25]. Notably, both T3 and high glucose concentrations are critical components of protocols that drive differentiation of stem cells to β-cells [14,[26], [27], [28]].

In mouse brown adipose tissue (BAT) we demonstrated that T3 and glucose synergistically regulate ChREBP, which in turnupregulates *Ucp1*, *Glut4* and *Fasn*, resulting in increased thermogenesis, decreased body weight, and improved glycemic levels. Recently, T3 was shown to promote lipogenesis in hepatocytes [30]. Similarly, T3 and glucose were shown to coordinately interact to activate ChREBPβ transcription, which in turn activates lipogenesis and fatty acid oxidation in hepatocytes [31]. In islets, both ChREBP splice isoforms- α & β [25], are expressed [29]. The expression of the β isoform is induced in response to increased glucose concentrations and is mostly nuclear, while ChREBPα is mostly cytoplasmic [25,32]. In β-cells, ChREBPβ (but not ChREBPα) expression is upregulated in response to glucose, leading to increased expression of known ChREBP target genes and increased β-cell proliferation [29]. Furthermore, this upregulation of ChREBPβ is required for glucose-stimulated β-cell proliferation and adaptive expansion of β-cell mass [29,32]. In pancreatic β-cells, ChREBP is a known regulator of liver-type pyruvate kinase (*Pklr*), which encodes an enzyme that catalyzes the conversion of phosphoenolpyruvate to pyruvate, the last step of glycolysis [33]. ChREBP also regulates expression of thioredoxin-interacting protein (*Txnip*) [34] which is involved in oxidative stress and is implicated in the regulation of β-cell death [35,36]. Other target genes of ChREBP include lipogenic genes, and hence ChREBP is thought to play a role in mediating glucolipotoxicity in β-cells [32,[37], [38], [39]]

Since ChREBP was shown to play a key role in glucose stimulated β-cell proliferation [29,40], we tested the hypothesis that glucose and T3 have a synergistic effect on ChREBP transcription and thus β-cell proliferation. We found that T3 and glucose act together to regulate expansion of β-cells in response to glucose. We identified a novel pathway that controls proliferation in pancreatic β-cells, the activation of phosphoenolpyruvate Carboxykinase (PEPCK-C) activity. PEPCK-C (gene name *PCK1*) is a main control point for the regulation of gluconeogenesis. PEPCK-C converts oxaloacetate and GTP into phosphoenolpyruvate, GDP and CO_2_. PEPCK promotes cancer cell proliferation *in vitro* and *in vivo* by increasing glucose and glutamine utilization toward anabolic metabolism. This effect is mediated at least partially by mTORC1 [41,42]. *PCK1* was demonstrated by Shalev et al. to be the second most glucose responsive gene in pancreatic human islets after *Txnip* [43]. In the liver, ChREBP is regulated by glucose levels [25,44], and also by T3 [45,46]. However, crosstalk or cooperative signaling effects between glucose and T3 in β-cells have not been studied.

While it is now established that human and murine α-cells express *PCK1* [47], it is widely thought that mature β-cells do not express *PCK1* [48]. In this study and by examining various available data sets for β-cell and human and rodent pancreatic progenitor cell differentiation, we found that PCK1 is expressed during maturation and development of β-cells [[49], [50], [51], [52], [53]], at a time when the proliferative capacity of β-cells is the highest [54,55]. We hence suggest a mechanism whereby T3 and glucose signaling pathways coordinately regulates transcription of genes important for β-cell function and mass, a novel concept in islet biology.

## 2 Materials and methods

### 2.1 Cell culture

INS-1–derived 832/13 rat insulinoma cells were maintained in RPMI 1640 medium with 10% FBS, 10 mM HEPES, 2 mM l-glutamine, 1 mM sodium pyruvate, and 50 mM β-mercaptoethanol, 100 U/mL penicillin, 100 mg/mL streptomycin and further supplemented with 11 mM glucose, at 37 °C in a 5% CO_2_ incubator. To specifically study the effect T3, 10% resin-stripped FCS, was used to deplete thyroid hormones as described in Cao et al. [56].

### 2.2 RNA-seq analysis

Total RNA from ∼100 islets per condition, from five different human donors was isolated using the RNAeasy micro kit (Qiagen) according to the manufacturer's protocol. RNA integrity was assessed using Ribogreen to determine total mass and Fragment Analyzer. All samples passed QC. The RQN (RNA quality) scores ranged from 7.7 to 10. Samples were submitted to the New York Genome Center and RNA was amplified via the NuGEN Ovation RNA-Seq System V2 prior to RNA sequencing. 35–40 million 2 × 50 bp paired-end reads were sequenced per sample on the HiSeq2500 instrument (Illumina). Raw count data was pre-filtered to keep genes with CPM >1.0 for at least 60% of the samples. After filtering, count data was normalized via the weighted trimmed mean of M-values [57] and normalized counts were further transformed into normally distributed expression values via the voom-transformation using a model that included technical and demographic covariates (gender, age, body mass index, intronic rate). We estimated the correlation between measurements made on the same subject using the limma function, duplicate Correlation and the intersubject correlation was input into the linear model fit using the limma block design [58]. The voom-transformed, adjusted expression data was the final input for statistical modeling. Statistical analysis was carried out using R language version 3.0.3 and its available packages [59]. Volcano plots were generated using ggplot2 function in R [60]. Data is available in GEO (GSE218334).

Comparisons between groups (log-fold-changes) were obtained as contrasts of the fitted linear modes generated using weighted least squares (lmFit) and empirical Bayes method [58,61]. A factorial design was also used to determine if genes respond differently to thyroid stimulation in low glucose versus high glucose concentrations (interaction term).

### 2.3 Identification of ChoREs

Carbohydrate response elements (ChoREs) binding motifs were downloaded from the Schmidt et al. paper [62], which aimed at determining such motifs by ChIP-seq in rat. By using the “seq2profile.pl” function of HOMER version 4.11 displayed in over the ChREBP chromatin peaks, we regenerated the ChoRE motif matrix used to build the top logo of Figure 3F from Schmidt et al. We then further “trained” the motif matrix by adding the ChoRE binding sites described by Poungvarin et al. [63] for mouse exons 1a and 1b. The final matrix (Supplementary Figure 9) was fed to the “findMotifs.pl” HOMER function by using the human GRCh38/hg38 and the GRCm38/mm10 mouse genomes. The coordinates of the ChoRE sites mapping within each of the genes (±5,000 bp) of Figure 5A and Supplementary Figure 8 were determined by using the “genome_join” function of the “fuzzyjoin” version 0.1.6 package of r 4.2.0.

### 2.4 THRB and RXRA sites

Coordinates of the binding sites for the human THRB and RXRA transcriptional regulators were downloaded from the ReMap2022 database (available at: https://remap.univ-amu.fr) [64]. For each transcriptional regulator, sites were mapped to the same gene area (±5,000 bp) as described above for the ChoREs.

Murine Thrb and Rxra transcriptional regulator binding sites were downloaded from, respectively, the Mendoza et al. [31] paper and the ReMap2022 database and mapped as above.

### 2.5 Pathway enrichment analysis of gene sets

Gene sets were tested for functional enrichment using the KEGG (downloaded 17/02/2020), Reactome (downloaded 17-02-2020) and Gene Ontology (downloaded: 03-04-2020) pathway databases using the Cytoscape (v3.7.2 PMID: 14597658) ClueGO (v2.5.7 PMID: 19237447) and CluePEDIA (v1.5.7 PMID: 23325622) apps. Pathways were reported with Benjamini–Hochberg (BH) multiple test correction >0.05. Gene sets were tested for transcription factor target enrichment using the GTRD (Gene Transcription Regulation Database v19.10 (GTRD, gtrd.biouml.org, [65]) collection from MSigDB [66] that was imported into the ClueGO environment. GTRD consists of genes predicted to contain transcription factor binding sites in their defined promoter region.

### 2.6 Immunostaining

After islet dispersal by 0.05% trypsin, cells were plated on 12-mm Laminin coated glass coverslips placed in 24-well plates (34,35). Islet cells were either uninfected or transduced with a multiplicity of infection (MOI) of 150 of the adenoviruses indicated. Thereafter, cells were incubated overnight in fresh medium with 10% strip FBS containing indicated glucose and T3 concentrations. Then, cells were rinsed with PBS and fixed in 4% paraformaldehyde, and β-cell proliferation by staining for ki67 (Thermo Scientific) and Insulin (Dako). At least 2000 β-cells were blindly counted per human donor/mouse. Cells were imaged on a Zeiss 510 NLO/Meta system (Zeiss, Oberkochen, Germany), using a Plan-Apochromat 20× objective.

### 2.7 Quantitative reverse transcription PCR

Total RNA was extracted using the Qiagen RNeasy micro kit, reverse transcription was performed using the MMLV reverse transcriptase (Promega), following by real-time PCR with the SYBER-green reagent (BioRad). The sequences of primers used are shown in Supplementary Table 1.

### 2.8 Chromatin Immunoprecipitation

Chromatin immunoprecipitation (ChIP) assays were performed with 100 mg of cell chromatin extracts from 20 × 10^6^ Ins1 cells. DNA was obtained with the Active Motif (Carlsbad, CA) chromatin shearing kit. Chromatin was precipitated by incubation with 3 μg of ChREBP antibody (Novus Biologicals) or 3 μg thyroid hormone receptor antibody which recognizes both THRA and THRB genes (Abcam, ab2743, clone C3 [67]) 1:10,000 dilution of rabbit immunoglobulin G (Abcam) followed by separation with protein G magnetic beads (Active Motif). Binding was analyzed by real-time PCR. Primer sequences are shown in Supplementary Table 1.

### 2.9 Proximity ligation assay (PLA)

PLA was used to determine endogenous protein–protein interactions [[68], [69], [70]]. ChREBP and ThR antibodies were conjugated to Duolink oligonucleotides, PLUS and MINUS oligo arms, respectively, using Duolink® In Situ Probemaker kits. Cells were rinsed with PBS, fixed with 4% methanol-free formaldehyde solution for 10 min at room temperature, and blocked with Duolink Blocking Solution for 1 h at 37 °C and then incubated with 4 μg/mL ChREBP-Plus and ThR-MINUS overnight at 4 °C. PLA was performed according to the manufacturer's directions. No secondary antibodies were used, because PLUS and MINUS oligo arms were directly conjugated to ChREBP and ThR. Cells were imaged on a Zeiss 510 NLO/Meta system (Zeiss, Oberkochen, Germany), using a Plan-Apochromat 63×/1.40 oil differential interference contrast objective.

### 2.10 Human islets

Human cadaveric islets received from the Integrated Islet Distribution Program were dispersed by trypsinization as described previously [19]. To obtain a population highly enriched in β-cells. Dispersed human islets were transduced with an adenovirus expressing ZsGreen driven by a MIP-miniCMV promoter and harvested by fluorescence-activated cytometric sorting (FACS Aria II) as described previously [71,72]. The β-cell fraction was confirmed to be >92% pure by immunolabeling of sorted cells with insulin, and by qRT-PCR [72].

### 2.11 Statistics

One-way or Two-way Anova was used to compare sets of data obtained from independent groups of samples. All data were analyzed using Prism version 9 (Graphpad software Inc., San Diego, CA). Statistical significance was considered at P < 0.05.

## 3 Results

### 3.1 Expression of ChREBP isoforms is dependent on both glucose and T3

**Figure 1:**
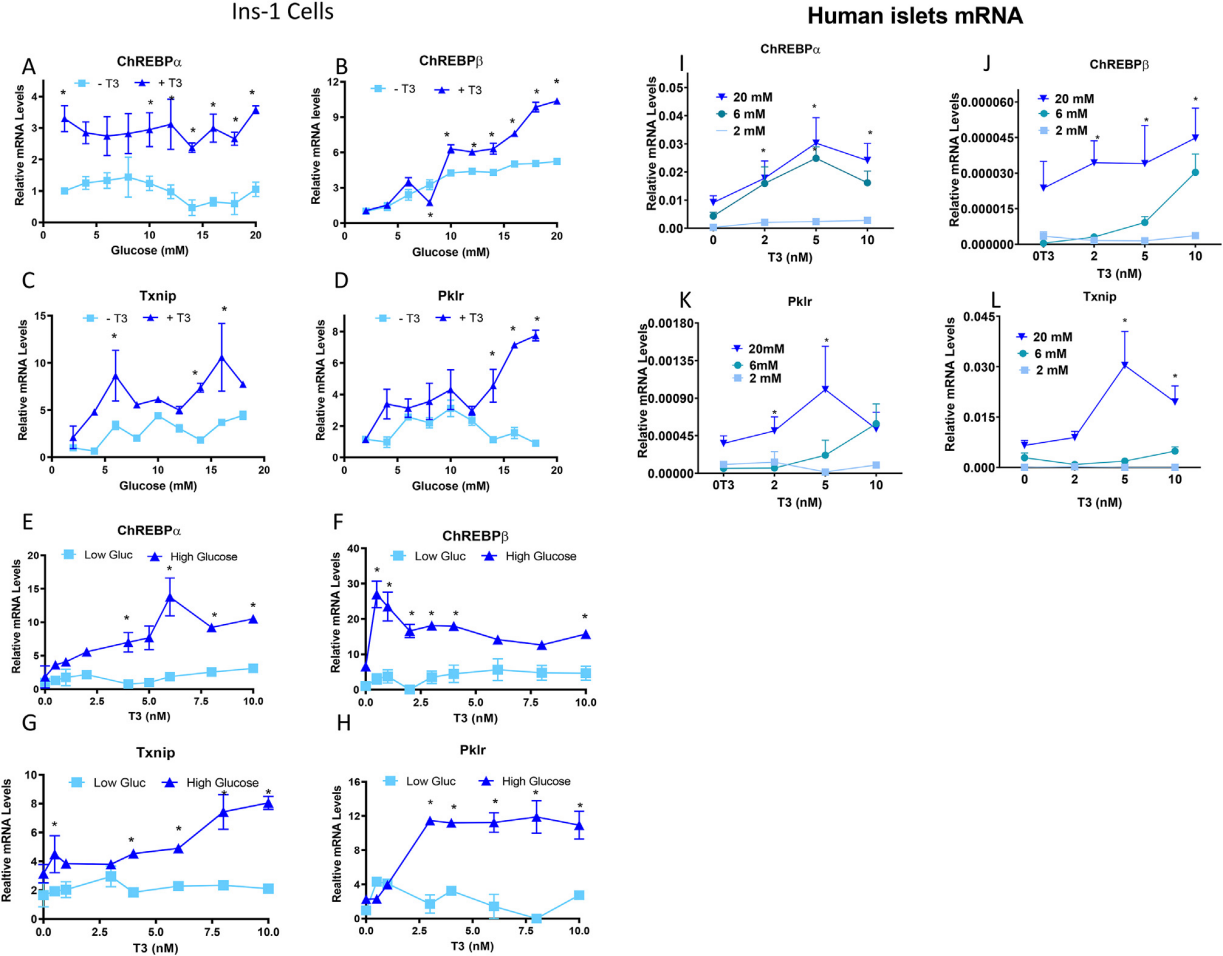
**ChREBP-dependent glucose responses require T3.** Ins-1 cells were cultured for 48 h in RPMI with 10% resin-stripped serum and the indicated concentrations of T3 and glucose. Response of ChREBPα (A, E), ChREBPβ (B, F), Txnip (C, G) and Pklr (D, H) mRNA levels to increasing glucose (A, D) or T3 (E, H) concentrations in cells incubated the presence (10 nM) or absence of T3 or in Low (2 mM) and High (20 mM) glucose. Data are the mean ± SEM of three independent experiments. (I–L) In human islets, ChREBPα (I) and Txnip (L) transcription is dose dependent on glucose concentration, while ChREBPβ (J) and Pklr (K) are dependent on T3 concentration. Human islets from five different, non-obese human donors were dispersed and cultured for 72 h in RPMI with 10% resin stripped serum. Islets were cultured in three different glucose concentrations (2, 6 and 20 mM) in combination with four different T3 concentrations (0, 2, 5 and 10 nM). mRNA was extracted and quantified by qPCR. Data are the means ± SEM of five independent experiments. All mRNA levels were normalized to β-actin; ∗P < 0.05 by two-way ANOVA.

To explore the relationship between T3 and glucose, we measured the expression of numerous genes following 48 h h of exposure to various concentrations of either glucose or T3 or a combination of both agents in INS-1 832/13 rat insulinoma cells [ [73] henceforth INS-1 cells]. Since fetal bovine serum contains relatively high concentrations of thyroid hormones, we utilized a T3/T4-free cell culture system by stripping FBS with anion exchange resin, which removes T3 and T4 from bovine serum [56]. We found that ChREBPα expression was induced in the presence of T3 but was not sensitive to changes in glucose concentrations (Figure 1A). By contrast, ChREBPβ expression was induced with increasing doses of glucose, in a dose-dependent manner both in the presence and absence of T3, which reached higher levels in the presence of T3 (10 nM), with the highest induction in the presence of T3 (Figure 1B). By comparison, in humans, according toAmerican Thyroid Association guidelines, the normal circulating levels of T3 are 0.9–2.8 nM and total T4 levels are 57–148 nM. When titrating T3 concentrations in either low (2 mM) or high (20 mM) glucose, we found that in ChREBPα expression was sensitive to changing T3 concentrations, but only in high glucose concentrations (Figure 1E). By contrast, ChREBPβ levels markedly increased with 1 nM T3 in high glucose but trended down with increasing concentrations of T3 (Figure 1F). ChREBP plays a number of important roles in pancreatic β-cells. In pancreatic β-cells, ChREBP is a known regulator of liver-type pyruvate kinase (Pklr), which encodes an enzyme that catalyzes the conversion of phosphoenolpyruvate to pyruvate, the last step of glycolysis [33]. ChREBP also induces expression of thioredoxin-interacting protein (Txnip) [34], which binds to and inhibits thioredoxin and is thus implicated in glucotoxic oxidative stress and β-cell death [35,36]. Other target genes of ChREBP include lipogenic genes as well as oxidative stress genes [74,75], thus ChREBP is thought to play a role in mediating glucolipotoxicity in β-cells [[37], [38], [39]]. Consistent with the changes in ChREBP expression, an effect of glucose concentration on the expression of the well-studied target genes of ChREBP genes, Pklr and Txnip was also noted. Txnip and Pklr expression increased in the presence of T3 (Figure 1C, D), and T3 potentiated the expression of these genes in high glucose (Figure 1G,H).

We next studied the effect of T3 and glucose concentrations on the expression of ChREBPα and β and the same target genes in human islets. Remarkably, we obtained very similar effects on mRNA expression in both model systems (Figure 1I–L). In the presence of T3 (2, 6 and 10 nM) the expression of ChREBPβ mRNA was highly responsive to varying concentrations of glucose. Transcription of ChREBPα in both 6 and 10 mM glucose was dose dependent on T3 levels. The responsiveness of ChREBP target genes TXNIP and PKLR showed a similar pattern of expression of ChREBPα and ChREBPβ to what was observed in Ins-1 cells (Figure 1). Together, these observations show a strong relationship between T3 and glucose signaling.

### 3.2 Knockdown of the thyroid hormone receptor results in downregulation of both ChREBP splice isoforms

**Figure 2:**
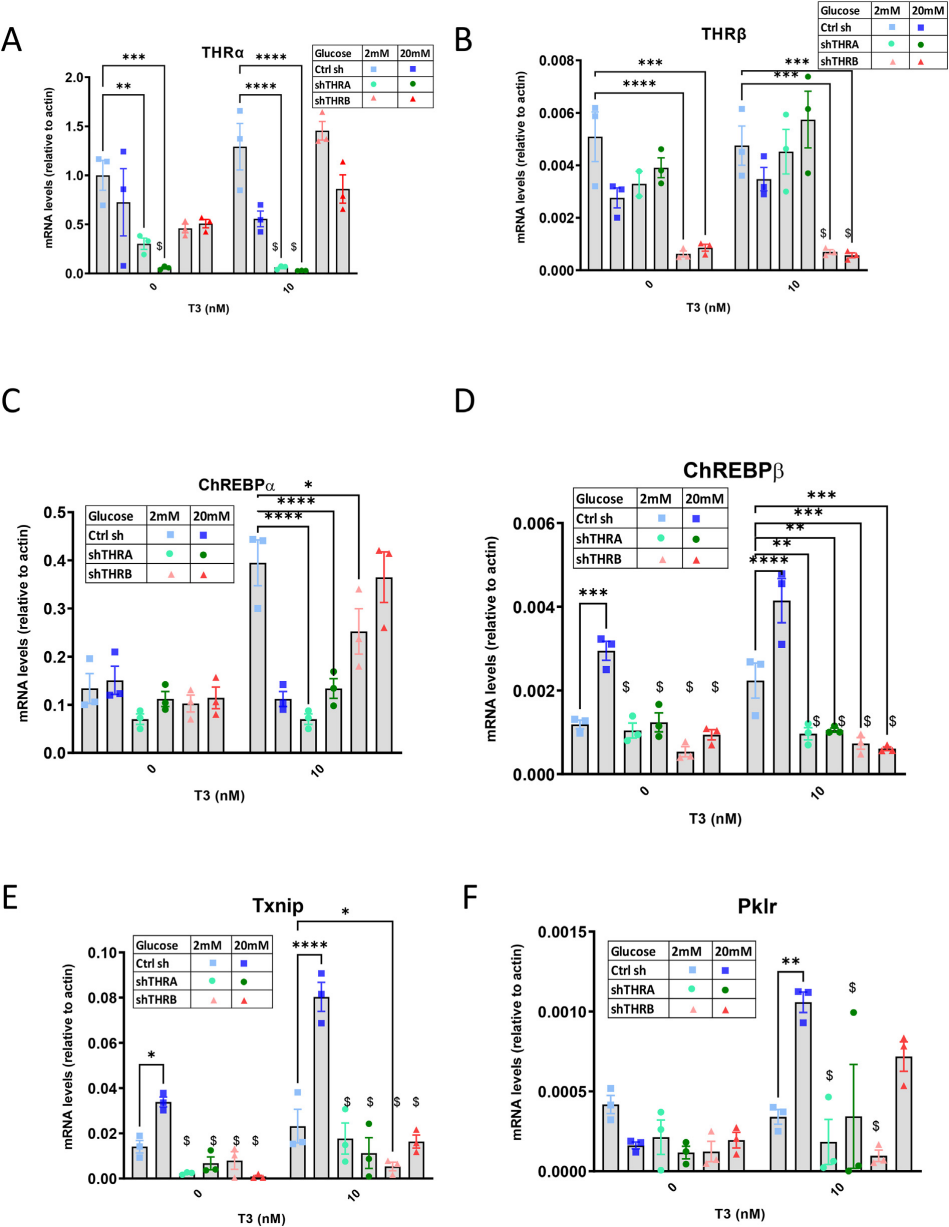
**Silencing of thyroid hormone receptors results in decreased ChREBPα and ChREBPβ transcription.** Ins-1 cells were transduced with lentivirus containing shRNA directed against Thra, Thrb or control shRNA. Following the transduction, Ins-1 cells were cultured for 48 h in RPMI with 10% resin stripped serum with the indicated glucose and T3 concentrations. Thra, Thrb, ChREBPα, and ChREBPβ mRNA levels were determined by qRT-PCR. (A, B) The specificity of each shRNA to silencing its own receptor was tested. Sequence for silencing as well as for qPCR detects both splice isoforms of each respective gene (C–F) The effect of knocking down each thyroid hormone receptors on ChREBPα (C) and ChREBPβ (D), Txnip (E), and Pklr (F) expression was examined. Data are the mean ± SEM of at least three independent experiments. All mRNA levels were normalized to β-actin.∗P < 0.05; ∗∗P < 001; ∗∗∗P < 005; ∗∗∗∗P < 001, compared to control 2 mM glucose within each respective group (0 nM T3 or 10 nM T3). $P < 0.05 compared to control 20 mM within each respective group. Statistical test-two way Anova.

Next, we tested whether silencing of the two thyroid hormone receptors (*Thra* and *Thrb*) would alter the expression of ChREBP. In rats, the two genes of Thr are expressed at different amounts during development. *Thra* is the predominate form just after birth in rodents. *Thra* and *Thrb* are expressed at equal levels from postnatal day 9–15, and after 15 days, *Thrb* becomes the predominant isoform in islets [12]. Here we find that in Ins-1 cells, *Thrb* is expressed at much higher levels than *Thra* [as can be appreciated by the respective mRNA levels compared to actin (Figure 2A,B)]. Using lentiviral shRNA, we silenced each of these genes in a specific manner (Figure 2A,B). Silencing of either Thra or Thrb resulted in a significant decrease in ChREBPα and ChREBPβmRNA levels (Figure 2C,D), with ChREBPβ decreased to similar levels by both THR isoforms shRNAs and ChREBPα decreased more efficiently with shThra. Txnip expression was efficiently repressed by both shRNAs (Figure 2E), whereas Pklr mRNA was decreased with shThrb only in the presence of T3. (Figure 2F).

### 3.3 Effect of T3 and glucose on beta-cell proliferation

**Figure 3:**
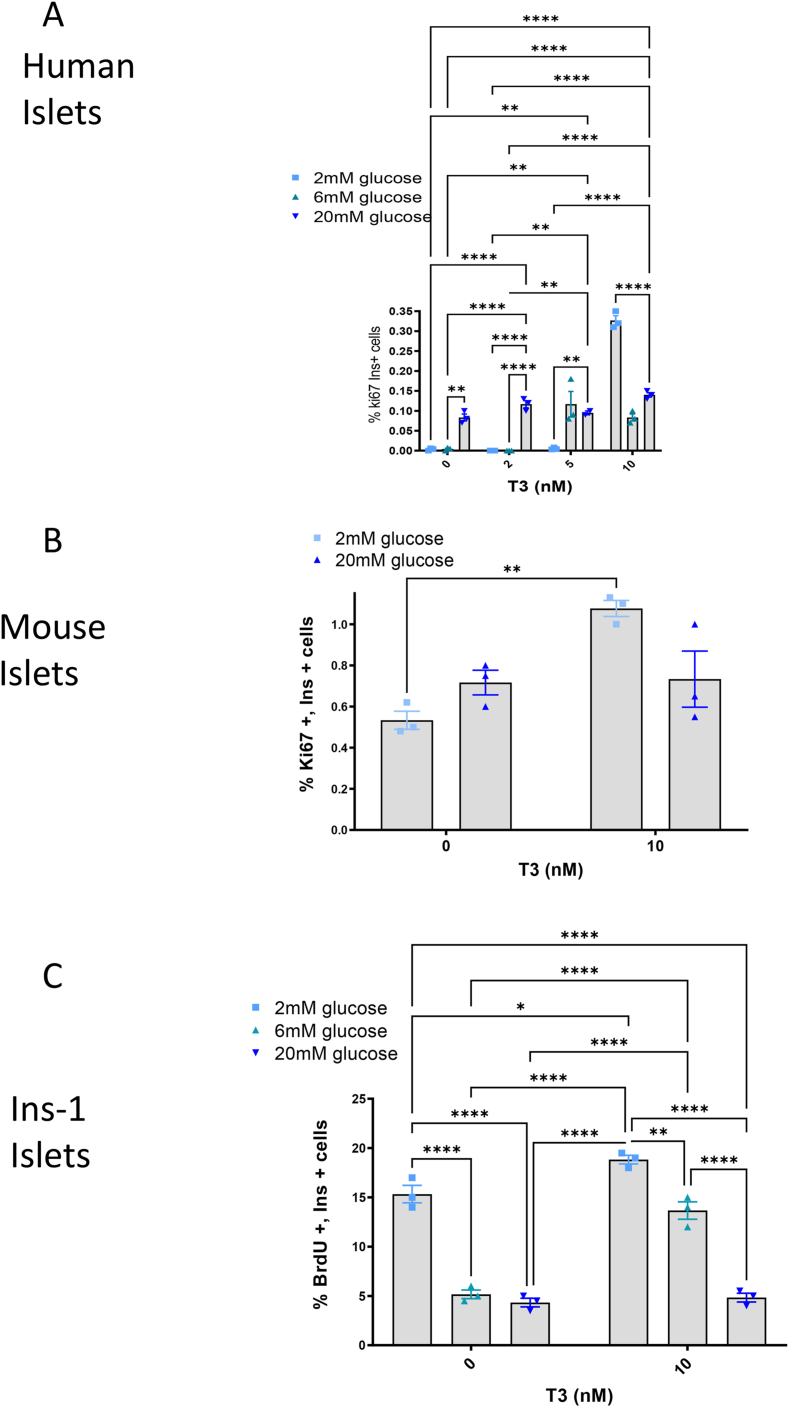
**T3 and glucose enhance β cell proliferation.** Human islets (A), Mouse islets (B) or Ins-1 cells (C) were dispersed and incubated at the indicated glucose or T3 concentrations in RPMI containing 10% resin-stripped serum. After 48 h, cells were fixed and immunolabeled for Ki67 and insulin. Presented are the percent of Ki67-positive and Insulin-positive cells. Data are the means ± SEM of at least three independent experiments. ∗P < 0.05; ∗∗P < 001; ∗∗∗P < 005; ∗∗∗∗P < 001 by two-way ANOVA.

Since ChREBP is essential for glucose-stimulated β-cell proliferation [29,32,74], we measured proliferation of β-cells (insulin positive cells) by Ki67 and insulin immunolabeling in isolated and dispersed human and mouse islet cells (Figure 3A,B), and BrdU immunostaining in Ins-1 cells (Figure 3C). In all three systems, glucose promoted proliferation, as expected (Supplemental Figure 1A-in human islets, visualized by the overall percent of cells positive for ki67). Yet, surprisingly, the highest percentage of cells that were double-positive for insulin and ki67 was obtained in low glucose and high T3 (Figure 3), indicating that fine tuning of glucose and T3 levels could be fundamental for controlling differentiation and proliferation of β-cells. It is therefore crucial to understand the mechanisms controlling expression of genes by those pathways and which genes are responsive to both T3 and glucose.

### 3.4 Genes upregulated by glucose and T3 in human islets

**Figure 4:**
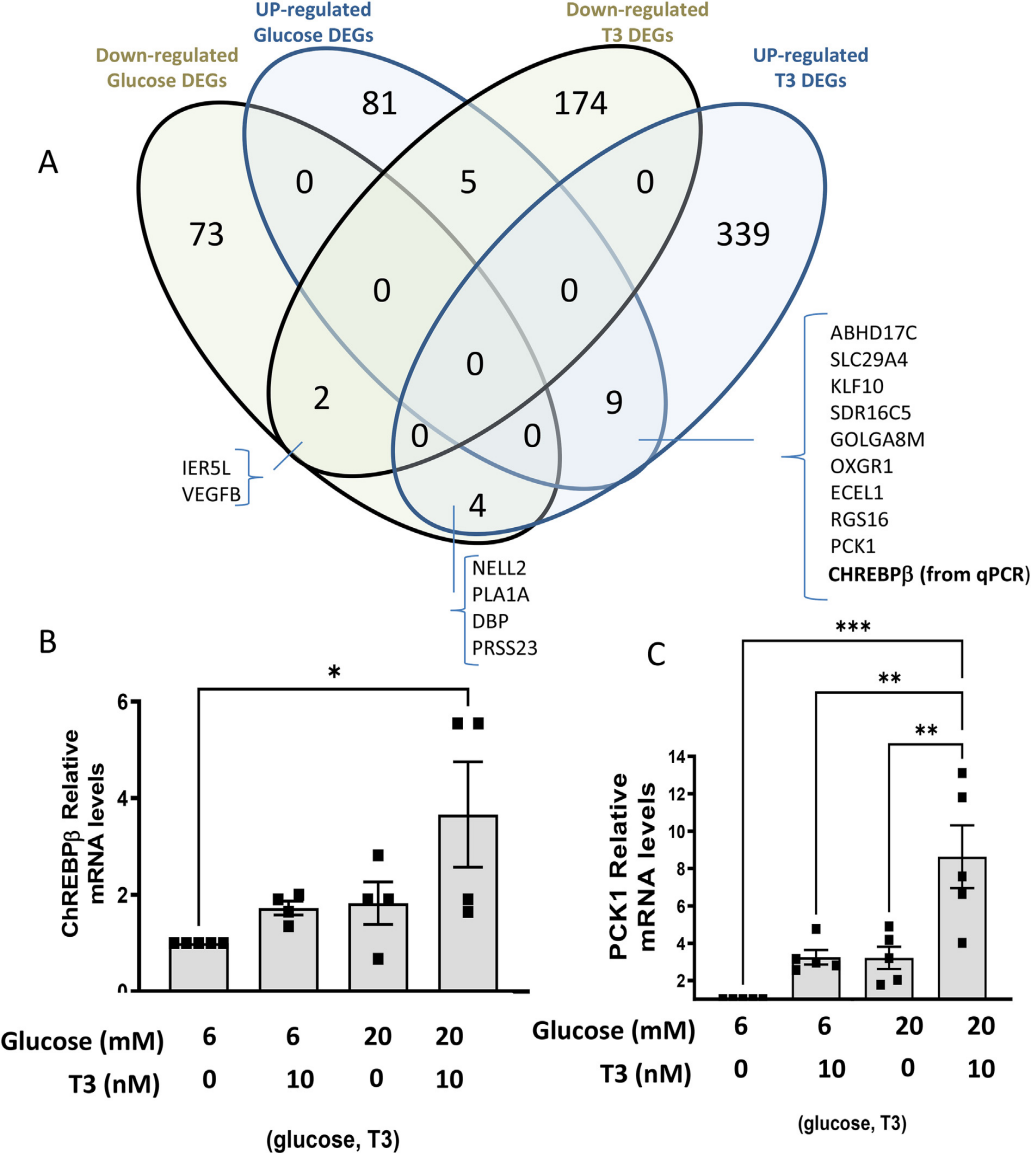
**Determining genes affected by high glucose and T3.** A. A Venn diagram showing the up- and down-regulated genes found differentially expressed either following glucose or T3 treatment. Only genes that were found significantly differentially expressed in either condition (at BH Adj P < 0.05 and no logFold change cut-off) were compared. Target validation, from the same donors used for the RNA-Seq, with indicated glucose and T3 concentrations. ChREBPβ and Pck1 mRNA levels were determined by qRT-PCR. Data are the means ± SEM of three independent experiments. All mRNA levels were normalized to β-actin. ∗P < 0.05; ∗∗P < 001; ∗∗∗P < 005 by one-way ANOVA.

We performed RNA-seq analysis of cadaveric human islets from five different donors in response to low and high glucose concentrations (6 and 20 mM, respectively) and in the presence or absence of T3 (10 nM). All donors were between the age of 24–61 and with body mass indexes (BMIs) ranging from between 18 and 26 (Supplementary Table 2). Covariate analysis was performed and BMI, intronic rate, age and gender were adjusted for and the multiple sampling from subjects was handled through the limma block function and duplicate correlation function (see Methods). We observed no significant interactions between the effect of glucose and T3 hormone on gene expression (Supplementary Figure 3). We therefore determined significantly differentially expressed genes (DEG) altered by glucose treatment regardless of T3 presence or altered by T3 regardless of glucose concentration. Volcano plots (Supplementary Figure 4) and a Venn diagram (Figure 4A) summarizing the intersection of the T3 (181 down- and 332 up-regulated genes) and high glucose responsive genes (91 down- and 73 up-regulated genes) are shown. Nine genes including PCK1 (phosphoenolpyruvate carboxykinase-1) were found commonly up-regulated by T3 or high glucose treatment, in addition to ChREBPβ, already identified by qPCR analysis (Figure 4A,B), which is a splice isoform of ChREBP that is highly glucose-responsive through a positive feed-back loop that promotes β-cell proliferation [25].

Pathway enrichment analyses of the DEGs associated with T3 and high glucose treatment are summarized in Supplementary Figures 5 and 6. Pathways associated with high glucose included ‘response to starvation’ and ‘amino acid regulation of mTORC1’. Pathways associated with T3 DEGs included ‘cellular response to hormone stimulus’ as well as ‘pancreatic secretion’ and ‘voltage-gated ion channel activity’. Transcription factor enrichment analysis of the genes upregulated by high glucose or T3 are shown in Supplementary Figure 7. Consistent with known glucose responsive elements, ChREBP-associated target genes were significantly enriched for in the high glucose DEGs, and THRA-associated target genes were significantly enriched for in the T3 up-regulated DEGs.

### 3.5 THR and ChREBP bind chromatin in close proximity

**Figure 5:**
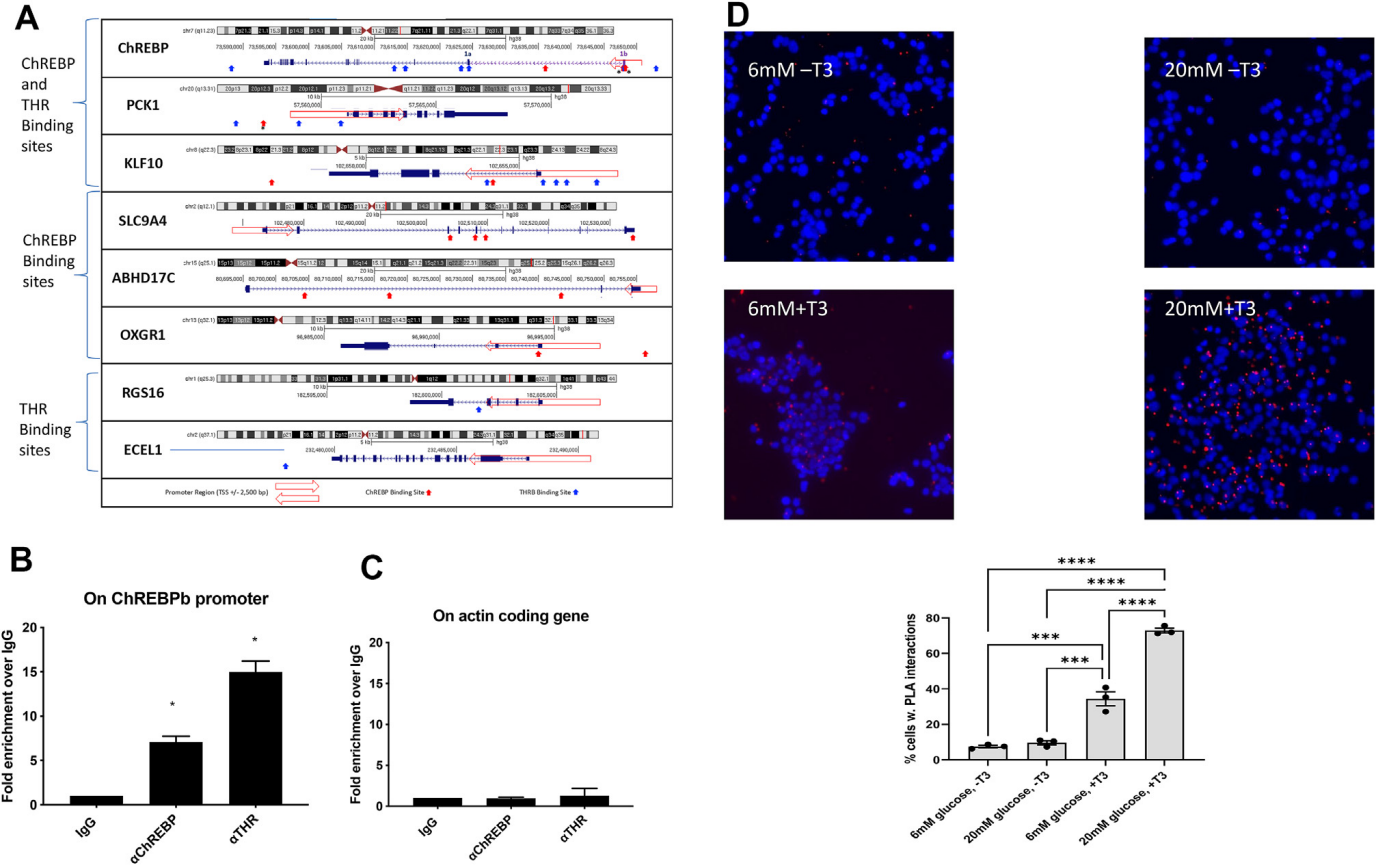
**Promoters of key regulatory genes for islet development contain THR and ChREBP binding sites.** A. ChREBP and THRB binding sites in human selected genes. Each panel is arranged as follows. The ideogram of the gene with its chromosomal location from the UCSC genome browser is shown. The representation displays exons (dark blue boxes) and introns (dark blue lines with arrowheads pointing to the direction of transcription). The promoter region (TSS ± 2,500 bp) is shown as a transparent red arrow. For the ChREBP gene, the position of the additional exon 1b is marked with a purple box and the intron between exons 1b and 1a is marked with a purple line with arrowheads oriented as for the rest of the gene. Blue and red upward arrowheads identify the center of ChREBP and THRB binding sites. ChREBP binding sites have been scored with the HOMER package (see material and methods) by using the frequency matrix of Supplementary Fig. 9, except for three sites that have been experimentally validated and are marked with asterisks near the respective arrowheads. The two ChREBP binding sites experimentally validated within exon 1b of the ChREBP gene have been tested by our lab. The single ChREBP binding site upstream of the PCK1 promoter has been tested by Jeong et al. [76]. THRB sites have been extracted from the ReMap2022 database. Supplementary Table 3 provides the coordinates of both ChREBP and THRB sites displayed. B. Chromatin Immunoprecipitation in Ins-1 cells grown in RPMI (11 mM glucose) supplemented with regular FCS. ChIP was performed using ChREBP and THR (alpha and beta, [30,67]) antibodies to detect binding on ChREBP promoter area and actin coding area (C). D. Proximity ligation assay for ChREBP and THR in Ins-1 cells was performed as described in materials and methods. Cells were growing low and high glucose, in the presence or absence of T3. Bottom panel-quantification of cells showing positive proximity ligation signal. ∗P < 0.05; ∗∗∗P < 0.01; ∗∗∗∗P < 0.005 using one-way ANOVA. (For interpretation of the references to color in this figure legend, the reader is referred to the Web version of this article.)

As a first approach to investigate cooperativity between T3 and glucose signaling, we concentrated on genes that are co-upregulated by both T3 and glucose-namely ChREBPβ, PCK1, SLC9A4, RGS16, ABHD17C OXGR1, KLF10 (Supplemental Figure 2). We identified ChREBP sites in the human genome by feeding to HOMER a carbohydrate response elements (ChoREs) binding site matrix (Supplementary Figure 9) obtained by using the ChoRE list from Schmidt et al. [62], the ChoRE sequences from Jeong et al. [76] and from our own experimental work on exon 1b of ChREBP (Figure 5A). To support our results, we conducted a parallel analysis with the mouse genome (Supplementary Figure 8). Binding sites for THRB were downloaded from the ReMap2022 database and Mendoza et al. [31] for human and mouse respectively (Figure 5, Supplementary Figure 8 and Supplementary Tables 3 and 4). We found biding sites for both ChREBP and THRs on promoters/gene regions of all genes in both human (Figure 5A) and mouse (Supplementary Figure 8). Interestingly, two genes were upregulated both by T3 and glucose in all four conditions tested in human islets, ChREBPβ and PCK1 (phosphoenolpyruvate carboxykinase-1). ChREBPβ is a splice isoform of ChREBP that is glucose responsive and regulates β-cell proliferation [25]. PCK1 is involved in hepatic gluconeogenesis and glycerolneogenesis in fat tissue but is not typically expressed in mature pancreatic β-cells [47]. Pck1 is a well-studied target gene of T3 in hepatocytes [77].

We identified conserved thyroid response element (TRE) and ChREBP binding sites in the promoter of the ChREBPβ isoform (Figure 5A and Supplementary Figure 8). We validated those positionson the ChREBPβ promoter that bind ChREBP and THR, respectively using ChIP (Figure 5B). We noticed some of the THR and ChREBP binding sites identified on the ChREBP promoters are in very close proximity with each other. Therefore, a proximity ligation assay (PLA) was performed to determine whether endogenous protein–protein interactions exist. A fluorescent signal is obtained when the distance is less than 40 nm between THR and ChREBP (Figure 5D). We found that in the presence of T3, both in low and high glucose, there is a physical interaction between these two transcription factors. These results suggest a cooperativity between these two transcription factors to integrate T3 and glucose signals.

### 3.6 PCK1 is regulated by glucose and T3 and its activity drives proliferation of β-cells

**Figure 6:**
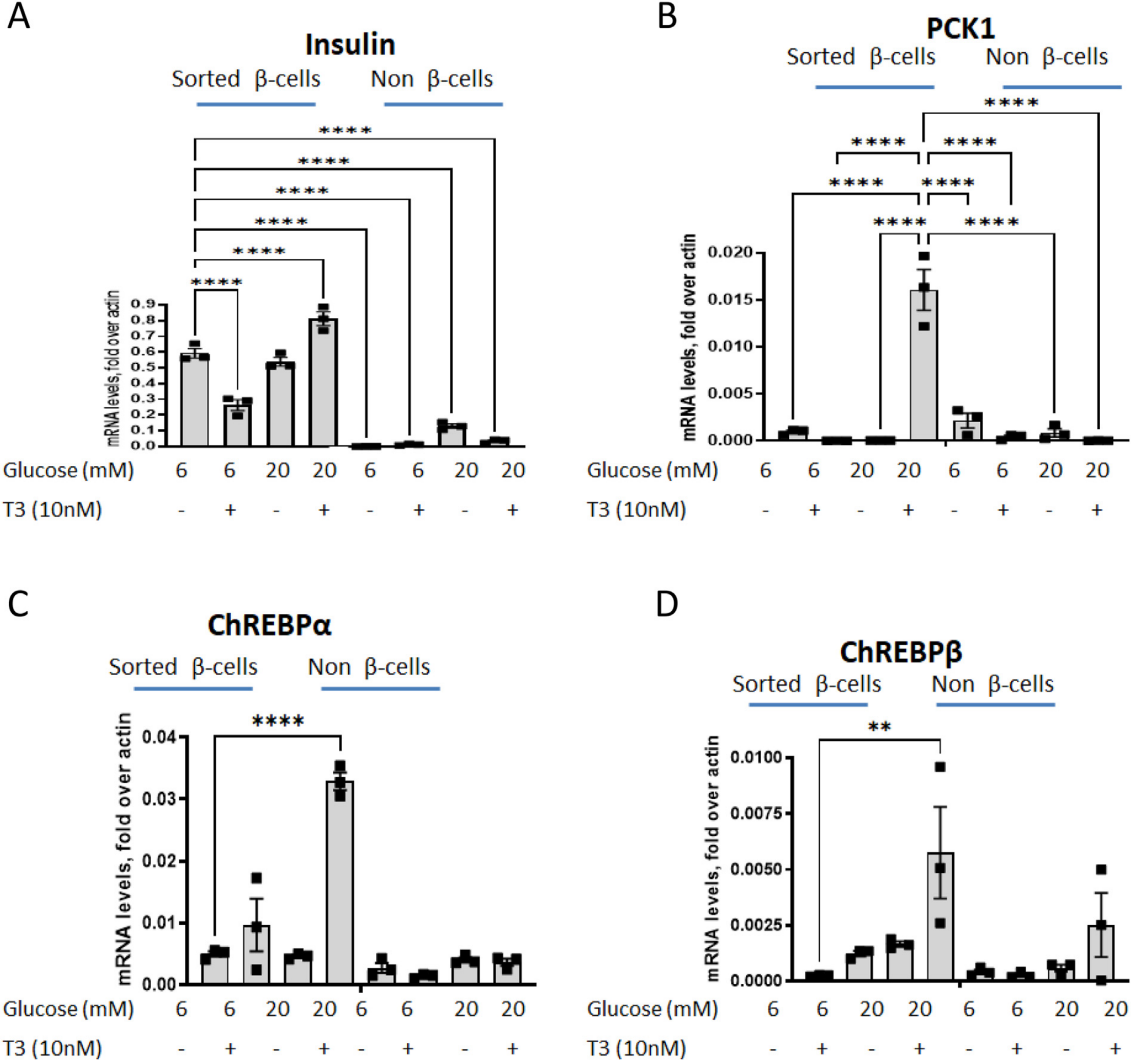
**PCK1 is expressed in human ß-cells exposed to high glucose and T3 concentrations.** Human islets were transduced with adenovirus expressing ZsGreen under the rat insulin promoter. Islets were dispersed and cultured in low (6 mM) or high (20 mM) glucose concentrations. After 48 h, cells were collected and sorted by FACS to separate β-cells from non-β-cells. mRNA was extracted and qPCR was performed to assess the levels of insulin, PCK1, ChREBPα or ChREBPβ. Data are the mean ± SEM of at least three independent experiments. ∗P < 0.05; ∗∗P < 001; ∗∗∗P < 005; ∗∗∗∗P < 001.

PCK1 is typically not expressed at high levels in mature β-cells. However, a recent study by Jaccovetti et al. comparing mRNA expression from young (p10) rats and adult rats, found that Pck1 is expressed 1000-fold higher in young rat islets compared with adults [50]. Similarly, Avrahami et al. recently found that Pck1 is expressed in beta cells of newborn humans [49]. Developmentally, β-cells proliferate at their highest rate just after birth [78,79]. We tested if combined treatment of T3 and glucose under our culture conditions would increase expression of PCK1 in human β-cells, and if any upregulation contributed to β-cell proliferation. Dispersed human islet cells were transduced with RIP-ZsGreen (as a marker to identify and sort β-cells [71]), treated with 6 mM or 20 mM glucose in the presence or absence of 10 nM T3 and cell-sorted to separate β-cells and non- β-cells. RNA was isolated and RT-qPCR was performed. Following sorting, we obtained a population of β-cells highly enriched in insulin mRNA (Figure 6A). Pck1 was highly upregulated with a combination of 20 mM glucose and 10 nM T3 In β-cells, but not in non- β-cells (Figure 6B). Additionally, 20 mM glucose and 10 nM T3 increased the expression of both ChREBPα and ChREBPβ exclusively in β-cells (Figure 6C,D). In additional, looking carefully at datasets available for islets on GDS browser (https://www.ncbi.nlm.nih.gov/sites/GDSbrowser), we are clearly able to demonstrate that Pck1 is expressed in rodent and human islets as well as in purified β-cells (Table 1).

**Table 1:** Summary of publicly available GDS datasets for pancreatic islets and purified β-cells suggests Pck1 is expressed in β-cells.

GEO profile	Organism	Citation	Pck1 in islets/β-cells	Comments
GDS4934	*Mus musculus*	[80]	β-cells	Pck1 is expressed in alpha, beta cells and beta cells from Pdx1 KO
GDS4935	*Rattus norvegicus*	[81]	β-cells	Pck1 is expressed in alpha, beta cells and β-cells from Pdx1 overexpression
GDS4937	*Rattus norvegicus*	[82]	β-cells	Pck1 is expressed at higher levels in 2-3 day neonates β-cells compared to 10 week adults
GDS4942 and GDS4939	*Mus musculus*	[83]	Islets	Pck1 is expressed in pancreatic islets young and adults mice cultured in low and high glucose
GDS4320	*Mus musculus*	[84]	Islets	Pck1 is expressed in control and PPARβ/δ
GDS4337	*Homo Sapiens*	[[85], [86], [87], [88]]	Islets	Pck1 is expressed in pancreatic islets from T2D and Control donors
GDS3983	*Homo Sapiens*	[89]	Islets	Pck1 is expressed in pancreatic endocrine cells, at lower levels compared with colon, kidney and small intestine
GDS3984	*Homo Sapiens*	[90]	Islets	Pck1 is expressed in islets, dedifferentiated and redifferentiated islets
GDS3991	*Mus musculus*	[91]	β-cells	Pck1 is expressed in β-cells at time of isolation as well as 24h and 48h past isolation
GDS4116	*Mus musculus*	[92]	β-cells	Pck1 is expressed in islets and in purified β-cells from Rag-/- mice
GDS5618	*Mus musculus*	[93]	Islets	Higher expression of Pck1 in islet graft
GDS4933	*Mus musculus*	[94]	Islets	Pck1 is expressed in conditional activation of IKK2/NF-κB in pancreatic beta-cells and control islets
GDS5380	*Mus musculus*	[95]	β-cells	Pck1 is expresses in control and Irs1 knock out islets with or without Tungstate.

**Figure 7:**
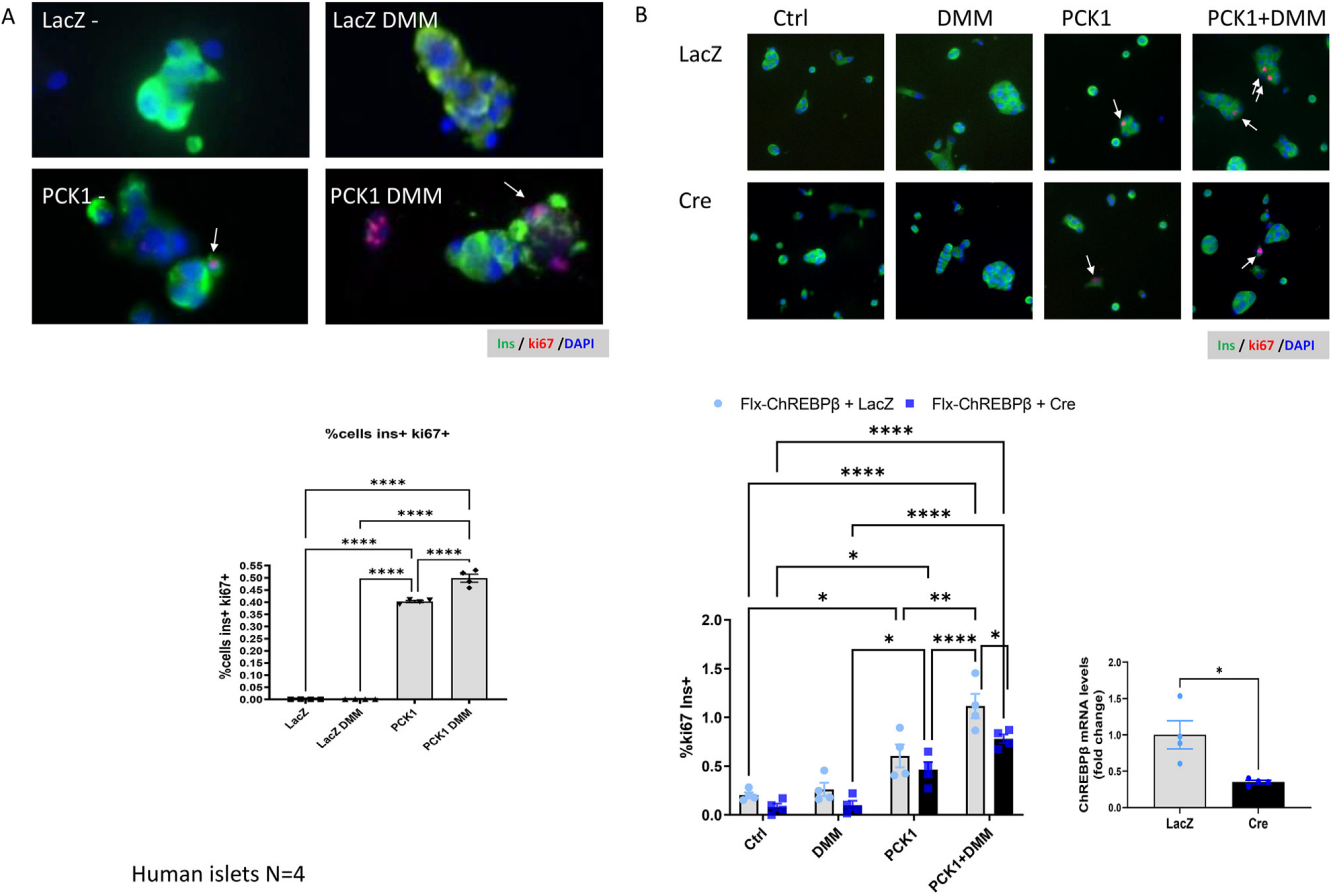
**PCK1 activity derives proliferation of ß-cells.** A. Human islets were transduced with an adenovirus containing PCK1 or control adenovirus (LacZ), in the presence, or absence of dimethyl malate (DMM, 10 mM). Dispersed islets were cultured in RPMI (5.5 mM glucose) with regular (therefore containing T3) 10% FCS. After 48 h, cells were fixed and stained with insulin and Ki67 to assess β-cell-specific proliferation. B. Isolated mouse islets from Floxed ChREBPβ mice were dispersed, cultured in RPMI (5.5 mM glucose) containing regular 10% FCS and transduced with LacZ, or Cre adenoviruses in the presence or absence of PCK1 Adenovirus and/or 10 mM DMM. Bottom right panel-mRNA levels of ChREBPβ from isolated islets from Floxed ChREBPBβ mice transduced with LacZ or Cre Adenovirus. Data are the means ± SEM of four independent experiments. ∗P < 0.05; ∗∗P < 001; ∗∗∗P < 005; ∗∗∗∗P < 001 by two-way ANOVA, or by student t-test for mRNA levels.

To test if PCK1 and its activity can control proliferation in β-cells, we overexpressed PCK1 in human islets cultured with non-stripped FCS and found that overexpression of PCK1 increases proliferation of adult human β-cells (Figure 7A). Furthermore, addition of dimethyl malate (DMM), a cell permeable substrate that can be metabolized to oxaloacetate, the substrate of Pck1, results in a significantly greater rate of β-cell proliferation (Figure 7A). Lastly, in mouse islets floxed for ChREBPβ, cultured with non-stripped FCS [32,74], we found significantly less proliferation when overexpressing PCK1 together with DMM in the absence of ChREBPβ, indicating that ChREBPβ is required for maximal proliferation in response to PCK1-overexpression (Figure 7B). Taken together we conclude that PCK1, whose expression is controlled by T3 and glucose, has the capacity to promote β-cell proliferation.

## 4 Discussion

In this paper, we demonstrate that thyroid hormone and glucose co-regulate ChREBP transcription and together the fine-tuning between the two signals can regulate gene expression and proliferation of rodent and human pancreatic β-cells. Our data indicate that the expression of ChREBPα is potentiated by T3, and that the expression of ChREBPβ and other downstream targets require and are augmented by T3. By stripping the serum in the growth media using resin we manage to eliminate thyroid hormone [56]. However, we also eliminate many other peptides and molecules that are important for β-cell proliferation and survival such as lactogens and growth factors. Therefore, we obtained lower proliferation rates than are normally found even when T3 is added back to the media, and it is likely that thi approach produces some alterations in gene expression and phenotype. However, the stripped-serum model system allows us to specifically study the role of T3 and in the context of ChREBP-dependent glucose-regulated gene expression, which plays important roles in glucose-stimulated β-cell proliferation and glucotoxicity [29,32]. T3 is a known regulator of endocrine cell maturation [11,12,96]. Our data demonstrates that in the absence of T3, in high glucose concentrations there were fewer insulin positive cells. Concurrently, more premature markers were starting to be expressed such as PCK1 and HR. Islets of newborn humans and newly born rats [49,50] express PCK1 shortly after birth. Similarly, PCK1 is expressed during the differentiation stages of embryonic stem cells, according to several data sets (see Table 1). While treatment with T3 is beneficial to patients with metabolic syndrome [[97], [98], [99]], the many side effects this drug has prevented it from being used in clinic. The observation that diabetes and thyroid dysfunction are closely linked is well-recognized [[1], [2], [3], [4], [5], [6], [7], [8], [9], [10]] and here we provide an insight as to how those two signaling pathways act together to regulate β-cell maturation and proliferation. While high T3 concentrations seem to lead to a less mature β-cell phenotype, low T3 concentrations would decrease proliferative capacity of β-cells, which might promote β-cell maturity on the one hand or prevent β-cell adaptation on the other providing hints to the comorbidity of diabetes and thyroid dysfunction. Yet, a β-cell specific THR agonist, similar to the one designed for liver to treat hyperlipidemia [[100], [101], [102]] could be developed to induce proliferation of β-cells as a potential therapeutic for both type 1 and type 2 diabetes where there is a deficiency in functional insulin producing cells.

Other genes that we found to be upregulated by T3 in both low and high glucose are Chodl, involved with carbohydrate sensing, enforcing the notion that T3 regulates glucose metabolism. Recently Ackerman et al. found Chodl (chondrolectin) to be one of the genes that is exclusively expressed in β-cells and not alpha cells [103] indicating T3 controls β-cell maturation. DBP was also found to be regulated by T3 in low and high glucose. DBP is involved in insulin production and secretion [104]. Polymorphisms in DBP are associated with Graves' disease and type 2 diabetes [105,106]. HR (hairless) is another one of the genes that is mostly regulated by T3 in both glucose concentrations tested. HR is a known target of thyroid hormone in the brain and skin and acts a transcriptional corepressor of the THR [107,108]. In skin and brain, it was also implicated in the regulation of cell proliferation [109]. As a member of the notch family, HR has also been demonstrated in pancreatic progenitors to control Hes1 expression, which in turn regulates the expression of Ngn3 [110]. We also found that CD14 was upregulated in islet cells and this molecule appears to be a functional LPS receptor on β cells [120]. In addition, we found several other genes whose roles in islet physiology are not fully understood. The genes that were responsive to glucose in the presence and absence of T3 are: Txnip, a major mediator of glucotoxicity [36]; Arrdc4, arrestin domain containing 4 that together with Txnip was identified to inhibit glucose uptake in adipocytes [121]; and RGS16, which controls differentiation of progenitors to islet cells [122]. These results are consistent with glucose being implicated both in islet-cell destruction and differentiation (Figure 3). As for the genes that we identified to be co-regulated both by glucose and by T3 (Figure 4 and Supplemental Figure 2), only a few recruit both ChREBP and THR to their proximal promoters and/or gene regions (namely ChREBP, PCK1, and KLF10 in both human and mouse as well as Abhd17c only in mouse). Yet in mouse, we found that all the co-regulated genes recruit ChREBP. Since the ChREBPβ promoter has binding sites for both thyroid hormone receptor and ChREBP, it is integrating both thyroid and glucose signaling, providing an insight for the mechanism of co-regulation. In addition, our data from the proximity ligation assay strongly suggests that with high T3 and glucose concentrations the two transcription factors are acting together in same complexes, and therefore suggest another possible insight for the co-regulation of downstream genes. Notably, the levels of the three deiodinase enzymes, important for the conversion of T4 to T3, remained unaltered in all conditions tested.

Cytosolic PCK1 is best known as a gluconeogenic enzyme, essential for the production of glucose in the liver in the fasted state [111,112]. PCK1 is also required for glyceroneogenesis in adipose tissue [113]. PCK1 is not expressed in mature β-cells, but it is apparent in databases of newborn islets [79], which corresponds developmentally with the time of greatest natural β-cell proliferation [54]. Several cancer cell lines have been described as having high expression of Pck1 that drive proliferation [41,114]. While the mechanism by which Pck1 influences increased proliferation is not fully understood, overexpression of PCK1 increases cataplerosis, allowing increased flux through the TCA cycle [115]. In addition, the production of PEP, the product of Pck1, may increase flux through the serine and nucleotide synthetic pathways. Since proliferating cells require increased carbon flux through these pathways [116], increased expression of Pck1 in non-gluconeogenic tissues may provide a metabolic solution for the requirement for increasing biomass. Interestingly, we observe that the induction of β-cell proliferation by PCK1 overexpression is not impaired by ChREBPβ deficiency. One possible explanation is that, similar to cancer cells, PCK1 may drive activation of mTORC1 and glucose utilization [41], which was previously described to induce proliferation of β-cells [117,118]. PCK1 also increases nucleotide synthesis and thus promotes proliferation in colorectal cancer cells [114], providing another possible mechanism for PCK1 mediated β-cell proliferation. We note that when adding DMM, the substrate for PCK1 we see that to achieve the highest β-cell proliferation, ChREBPβ is required.

In summary, T3 is necessary for glucose-mediated transcription in rodent and human βcells. T3 and glucose together upregulate Pck1, which is sufficient to drive βcell proliferation. Finding a mechanism and link between thyroid disorders and diabetes could help predict, prevent, and possibly treat diabetes. In the long term, ChREBP may be a target for therapeutic regulation of β-cell function, proliferation and survival. Additionally, a T3 analog with islet-selective activity could be designed, similar to the liver-specific thyroid hormone analog developed for the treatment of hyperlipidemia [119], and thus regulate glucotoxicity and β-cell mass. The mechanism by which Pck1 drives β-cell proliferation should studied in more detail, as it may provide unique pathways to therapeutically increase β-cell mass.

## Data Availability

Data will be made available on request.
