# Supplementary material for: T3 and glucose increase expression of phosphoenolpyruvate carboxykinase (PCK1) leading to increased β-cell proliferation
Source: Mol Metab. 2022 Nov 29;66:101646. doi: 10.1016/j.molmet.2022.101646 (PMC9731891; doi:10.1016/j.molmet.2022.101646)
Supplement: Multimedia component 1 [file mmc1.pptx]

## Slide 1
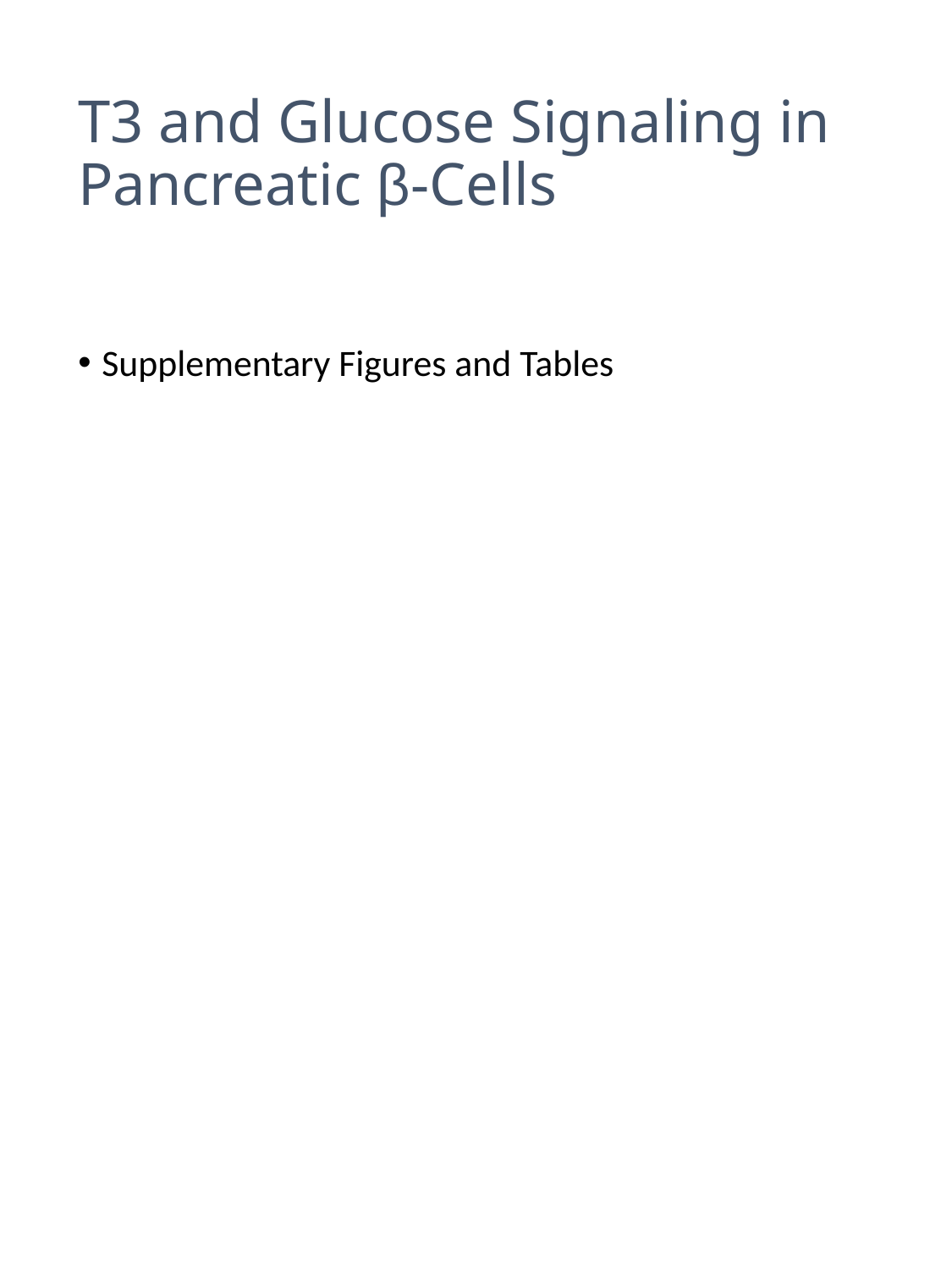

# T3 and Glucose Signaling in Pancreatic β-Cells
Supplementary Figures and Tables

## Slide 2
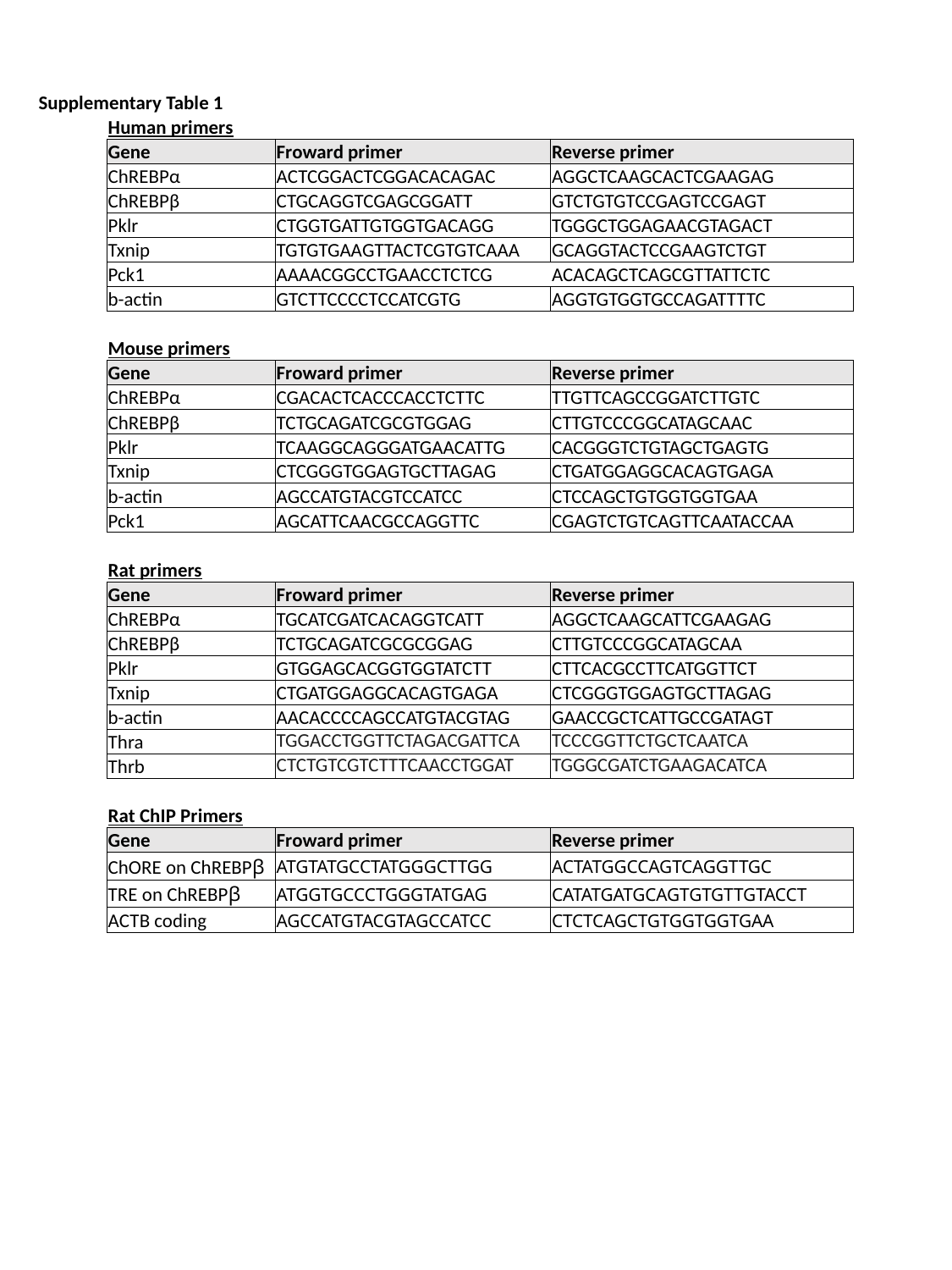

| Supplementary Table 1 | | | |
| --- | --- | --- | --- |
| | Human primers | | |
| | Gene | Froward primer | Reverse primer |
| | ChREBPα | ACTCGGACTCGGACACAGAC | AGGCTCAAGCACTCGAAGAG |
| | ChREBPβ | CTGCAGGTCGAGCGGATT | GTCTGTGTCCGAGTCCGAGT |
| | Pklr | CTGGTGATTGTGGTGACAGG | TGGGCTGGAGAACGTAGACT |
| | Txnip | TGTGTGAAGTTACTCGTGTCAAA | GCAGGTACTCCGAAGTCTGT |
| | Pck1 | AAAACGGCCTGAACCTCTCG | ACACAGCTCAGCGTTATTCTC |
| | b-actin | GTCTTCCCCTCCATCGTG | AGGTGTGGTGCCAGATTTTC |
| | | | |
| | Mouse primers | | |
| | Gene | Froward primer | Reverse primer |
| | ChREBPα | CGACACTCACCCACCTCTTC | TTGTTCAGCCGGATCTTGTC |
| | ChREBPβ | TCTGCAGATCGCGTGGAG | CTTGTCCCGGCATAGCAAC |
| | Pklr | TCAAGGCAGGGATGAACATTG | CACGGGTCTGTAGCTGAGTG |
| | Txnip | CTCGGGTGGAGTGCTTAGAG | CTGATGGAGGCACAGTGAGA |
| | b-actin | AGCCATGTACGTCCATCC | CTCCAGCTGTGGTGGTGAA |
| | Pck1 | AGCATTCAACGCCAGGTTC | CGAGTCTGTCAGTTCAATACCAA |
| | | | |
| | Rat primers | | |
| | Gene | Froward primer | Reverse primer |
| | ChREBPα | TGCATCGATCACAGGTCATT | AGGCTCAAGCATTCGAAGAG |
| | ChREBPβ | TCTGCAGATCGCGCGGAG | CTTGTCCCGGCATAGCAA |
| | Pklr | GTGGAGCACGGTGGTATCTT | CTTCACGCCTTCATGGTTCT |
| | Txnip | CTGATGGAGGCACAGTGAGA | CTCGGGTGGAGTGCTTAGAG |
| | b-actin | AACACCCCAGCCATGTACGTAG | GAACCGCTCATTGCCGATAGT |
| | Thra | TGGACCTGGTTCTAGACGATTCA | TCCCGGTTCTGCTCAATCA |
| | Thrb | CTCTGTCGTCTTTCAACCTGGAT | TGGGCGATCTGAAGACATCA |
| | | | |
| | Rat ChIP Primers | | |
| | Gene | Froward primer | Reverse primer |
| | ChORE on ChREBPβ | ATGTATGCCTATGGGCTTGG | ACTATGGCCAGTCAGGTTGC |
| | TRE on ChREBPβ | ATGGTGCCCTGGGTATGAG | CATATGATGCAGTGTGTTGTACCT |
| | ACTB coding | AGCCATGTACGTAGCCATCC | CTCTCAGCTGTGGTGGTGAA |

## Slide 3
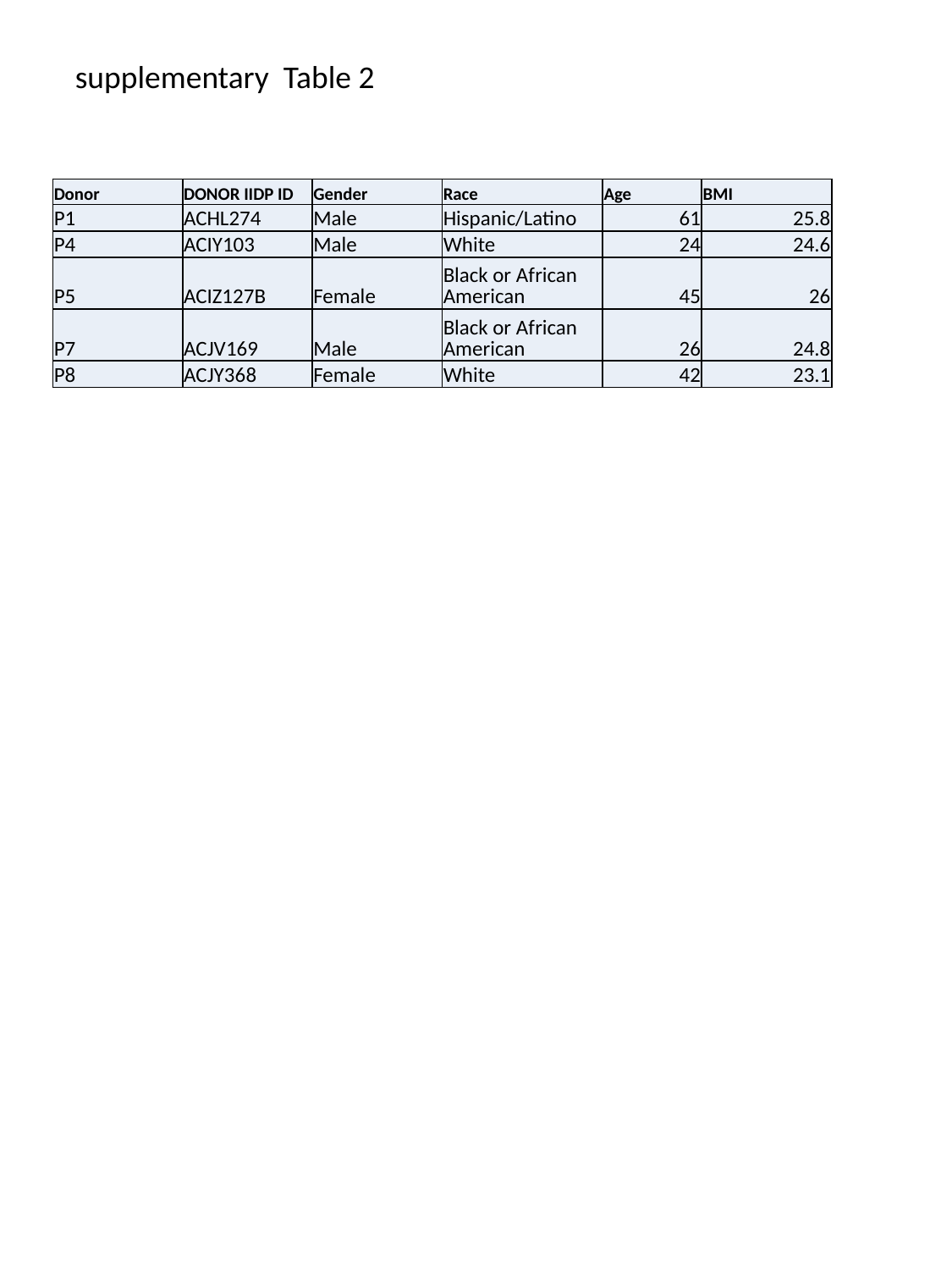

supplementary Table 2
| Donor | DONOR IIDP ID | Gender | Race | Age | BMI |
| --- | --- | --- | --- | --- | --- |
| P1 | ACHL274 | Male | Hispanic/Latino | 61 | 25.8 |
| P4 | ACIY103 | Male | White | 24 | 24.6 |
| P5 | ACIZ127B | Female | Black or African American | 45 | 26 |
| P7 | ACJV169 | Male | Black or African American | 26 | 24.8 |
| P8 | ACJY368 | Female | White | 42 | 23.1 |

## Slide 4
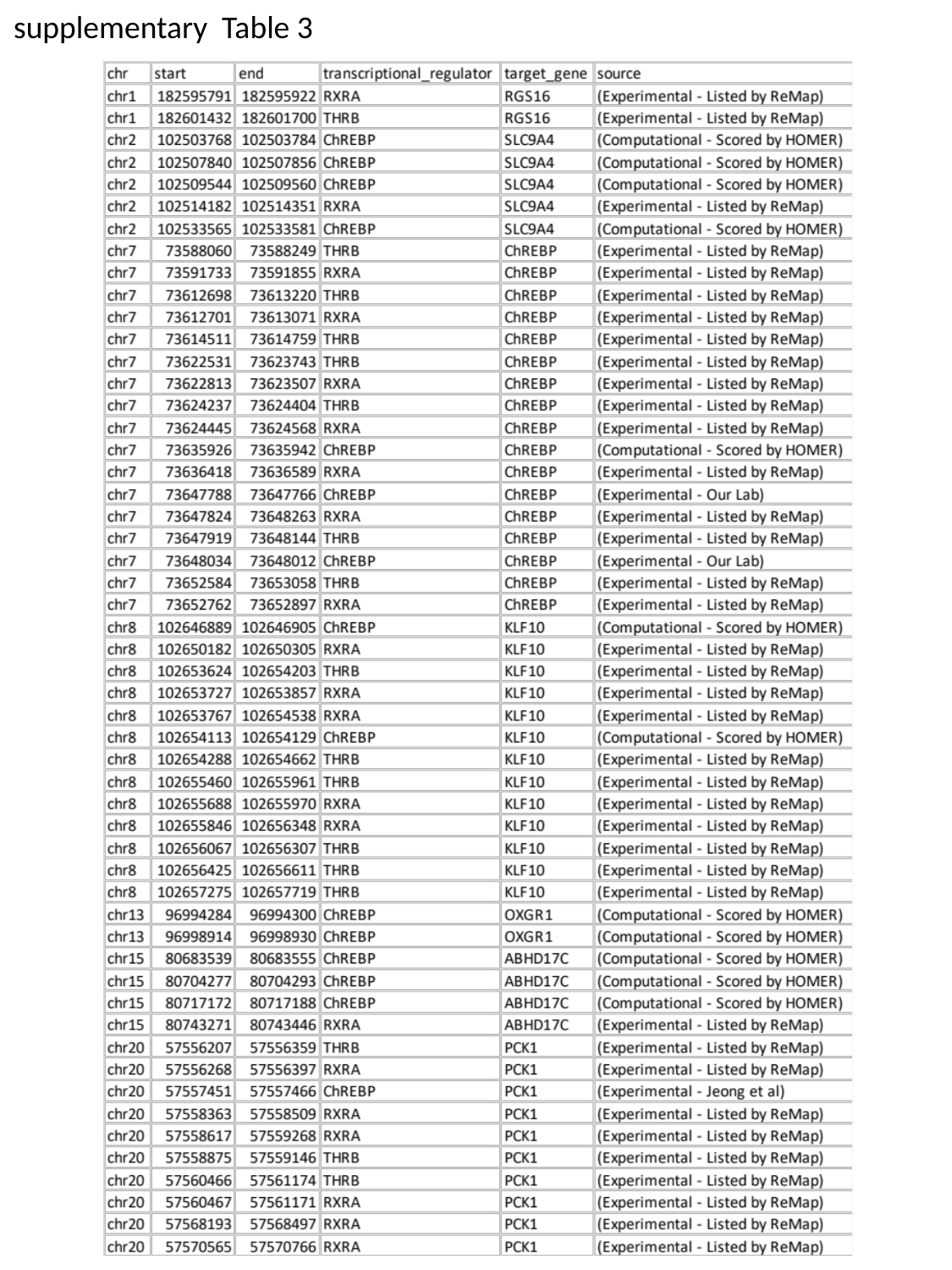

supplementary Table 3

## Slide 5
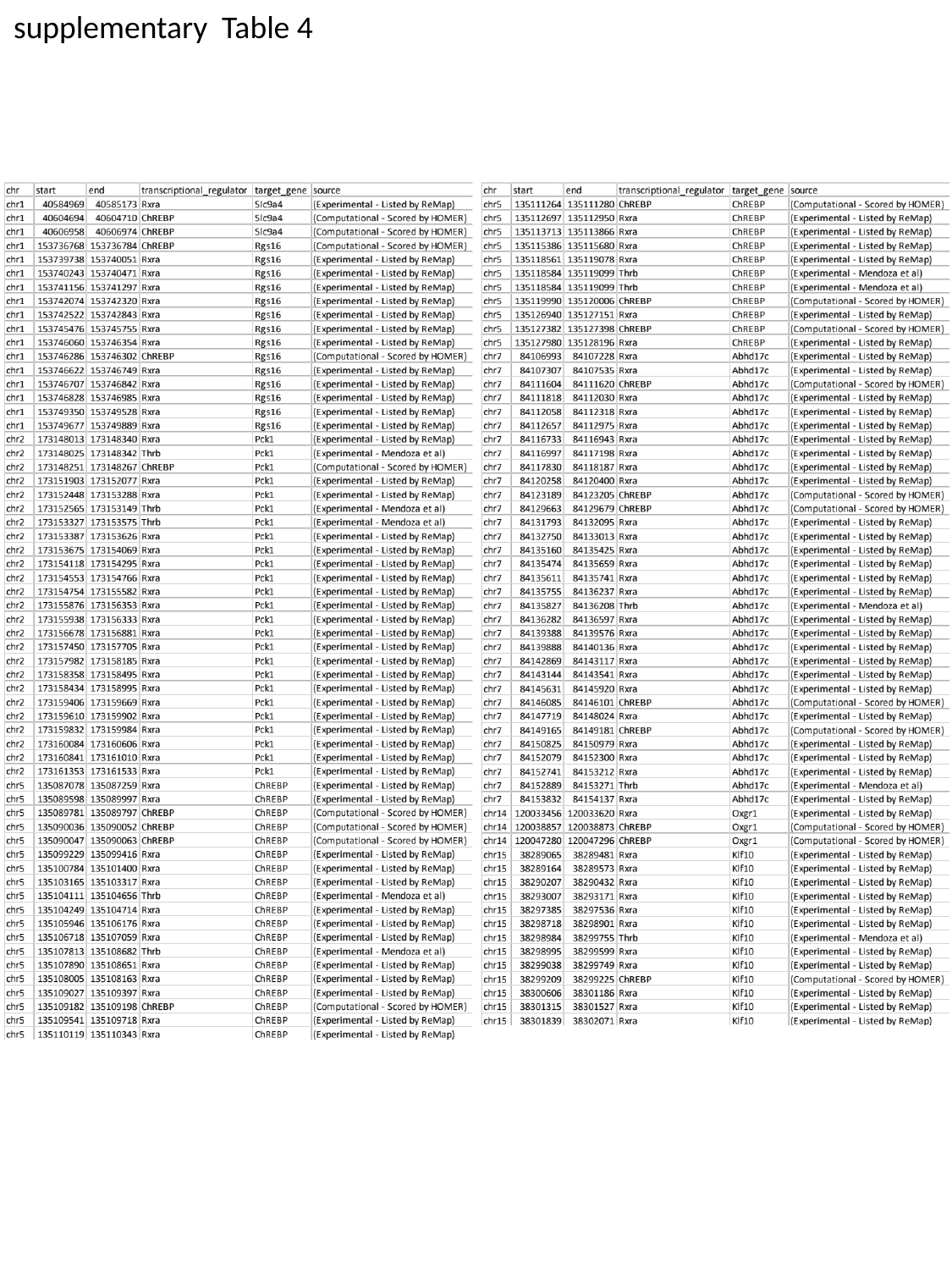

supplementary Table 4

## Slide 6
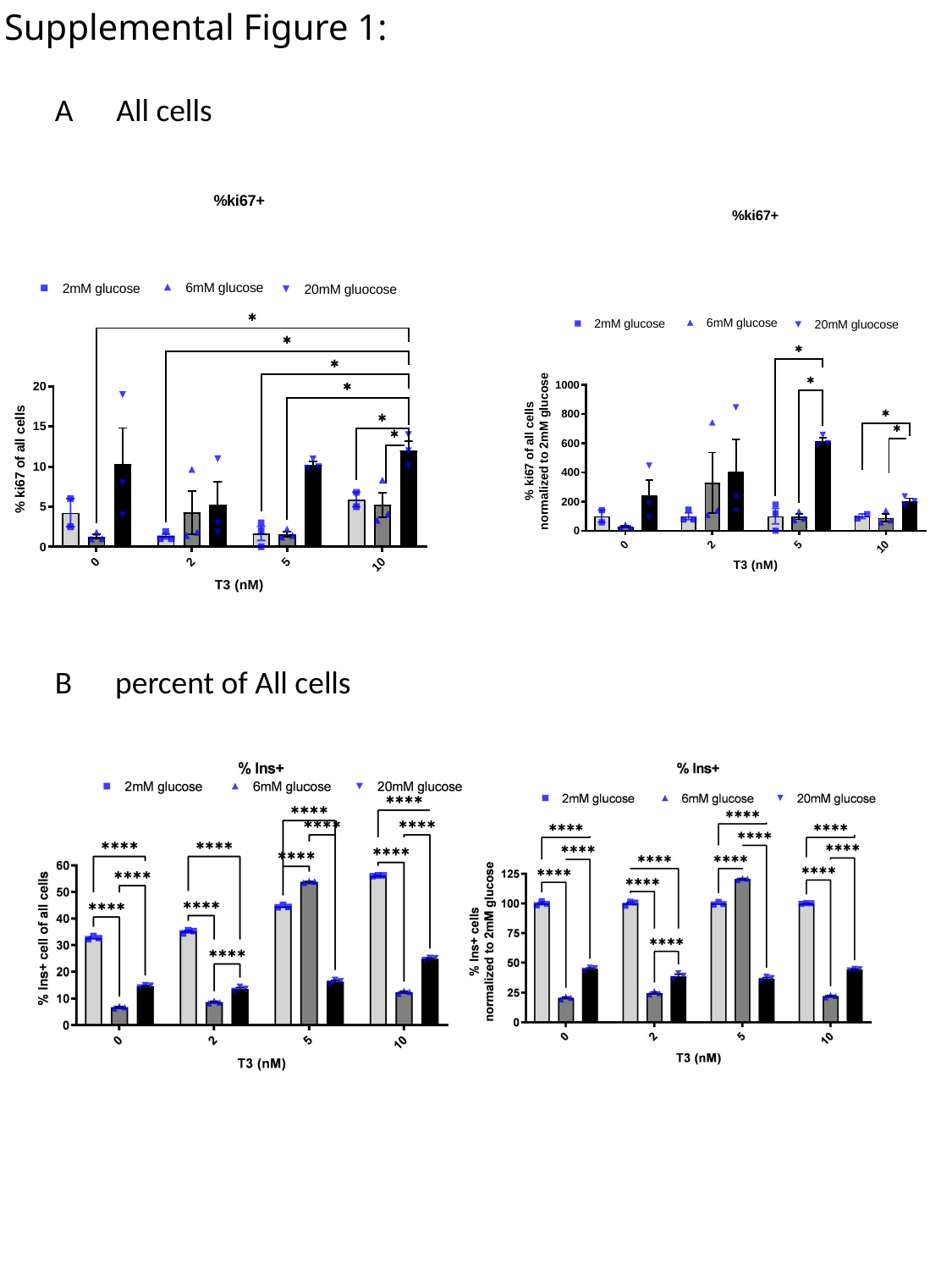

# Supplemental Figure 1:
A All cells
B percent of All cells

## Slide 7
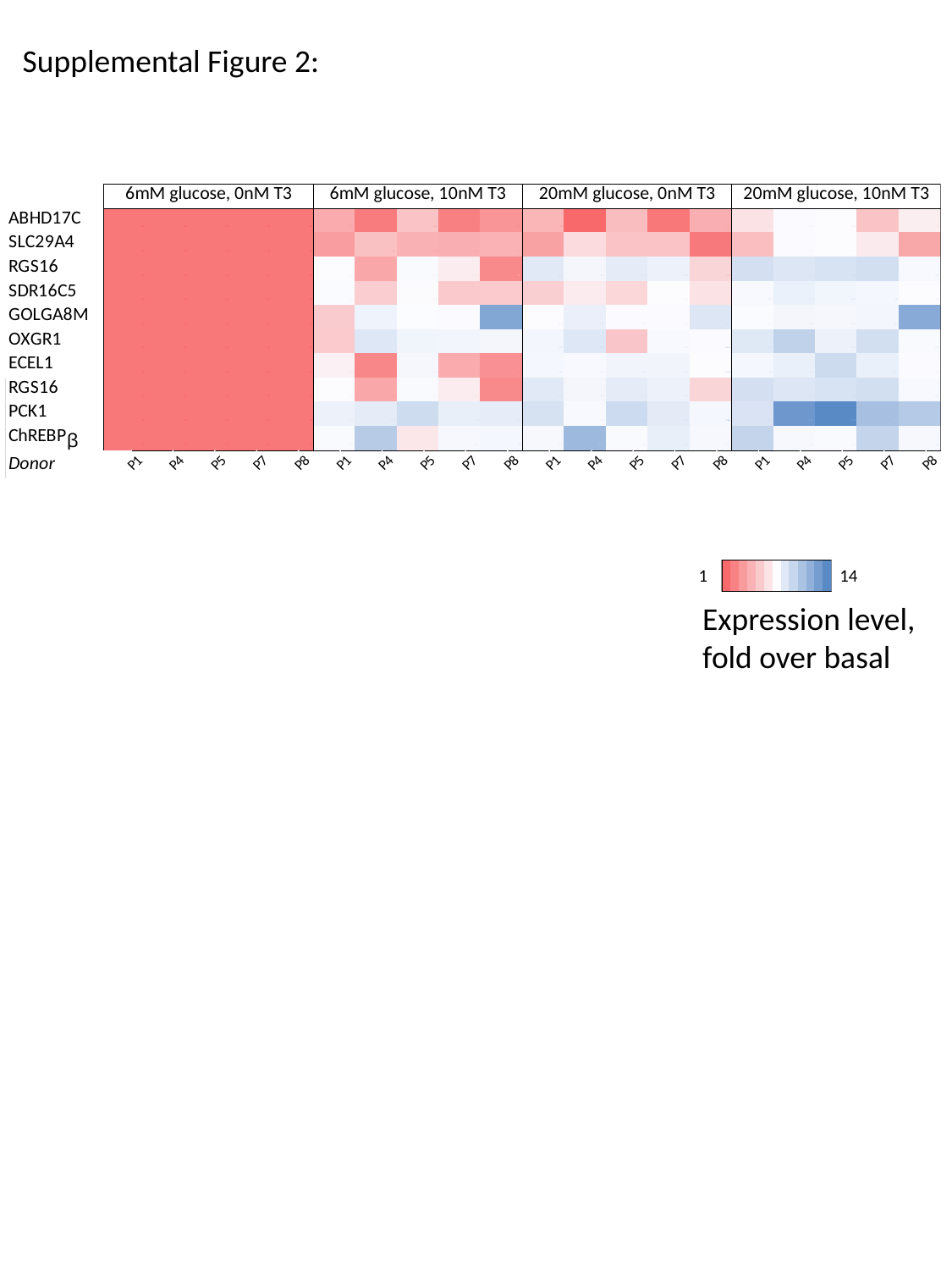

Supplemental Figure 2:
1 14
Expression level, fold over basal

## Slide 8
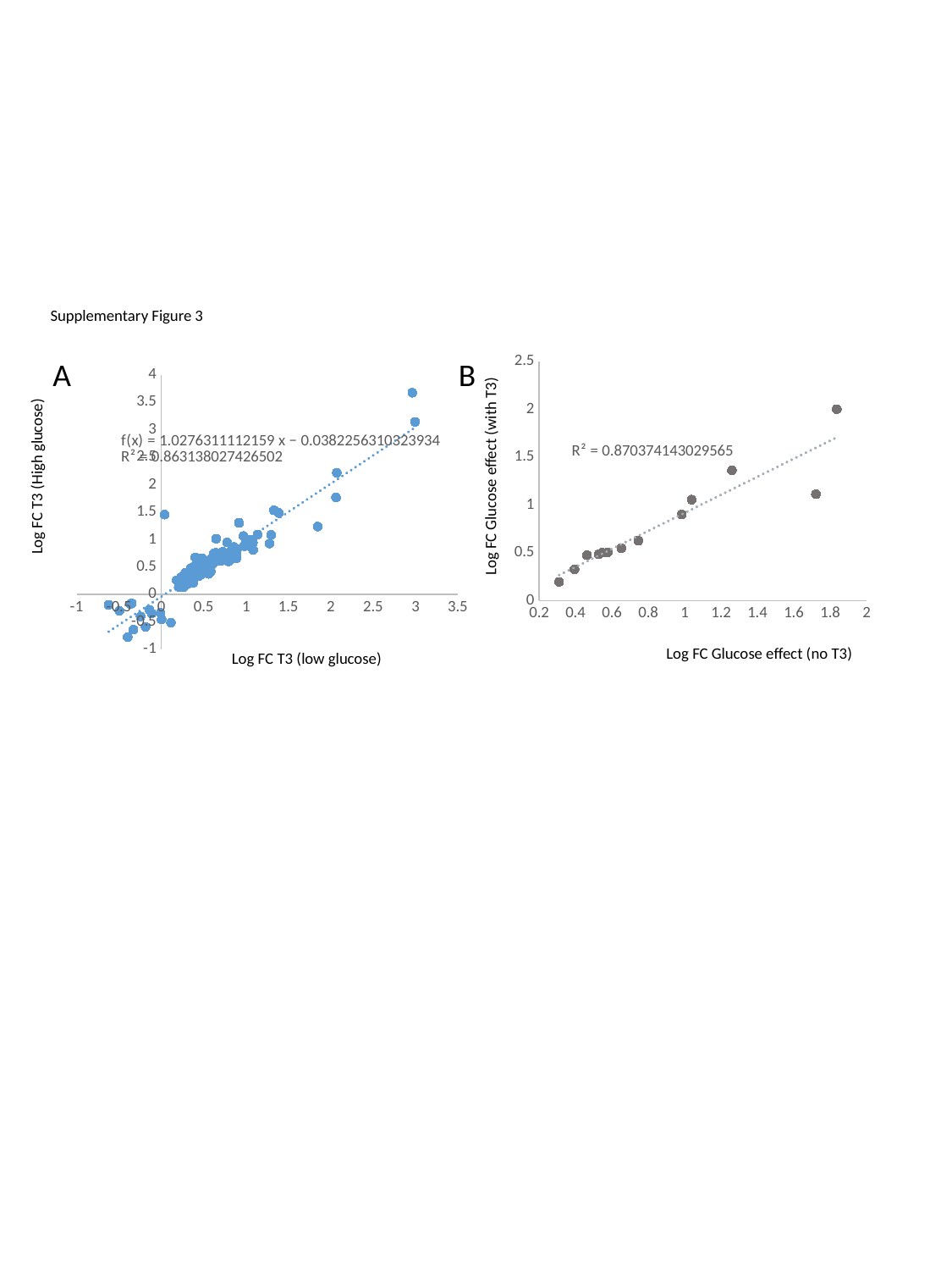

Supplementary Figure 3
A B
### Chart
| Category | |
|---|---|
### Chart
| Category | logFC.coef3 |
|---|---|Log FC Glucose effect (with T3)
Log FC T3 (High glucose)
Log FC Glucose effect (no T3)
Log FC T3 (low glucose)

## Slide 9
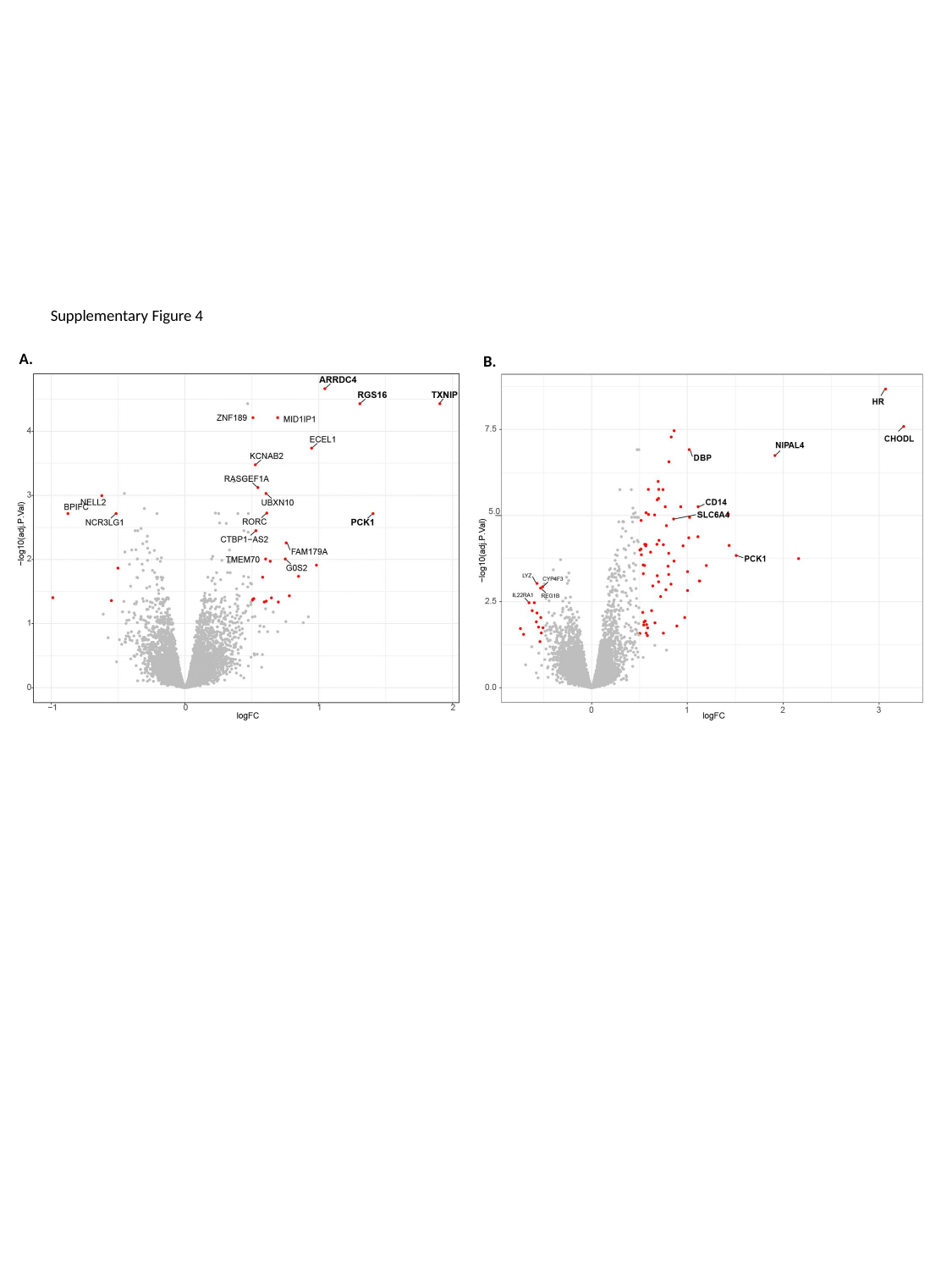

Supplementary Figure 4
A.
B.

## Slide 10
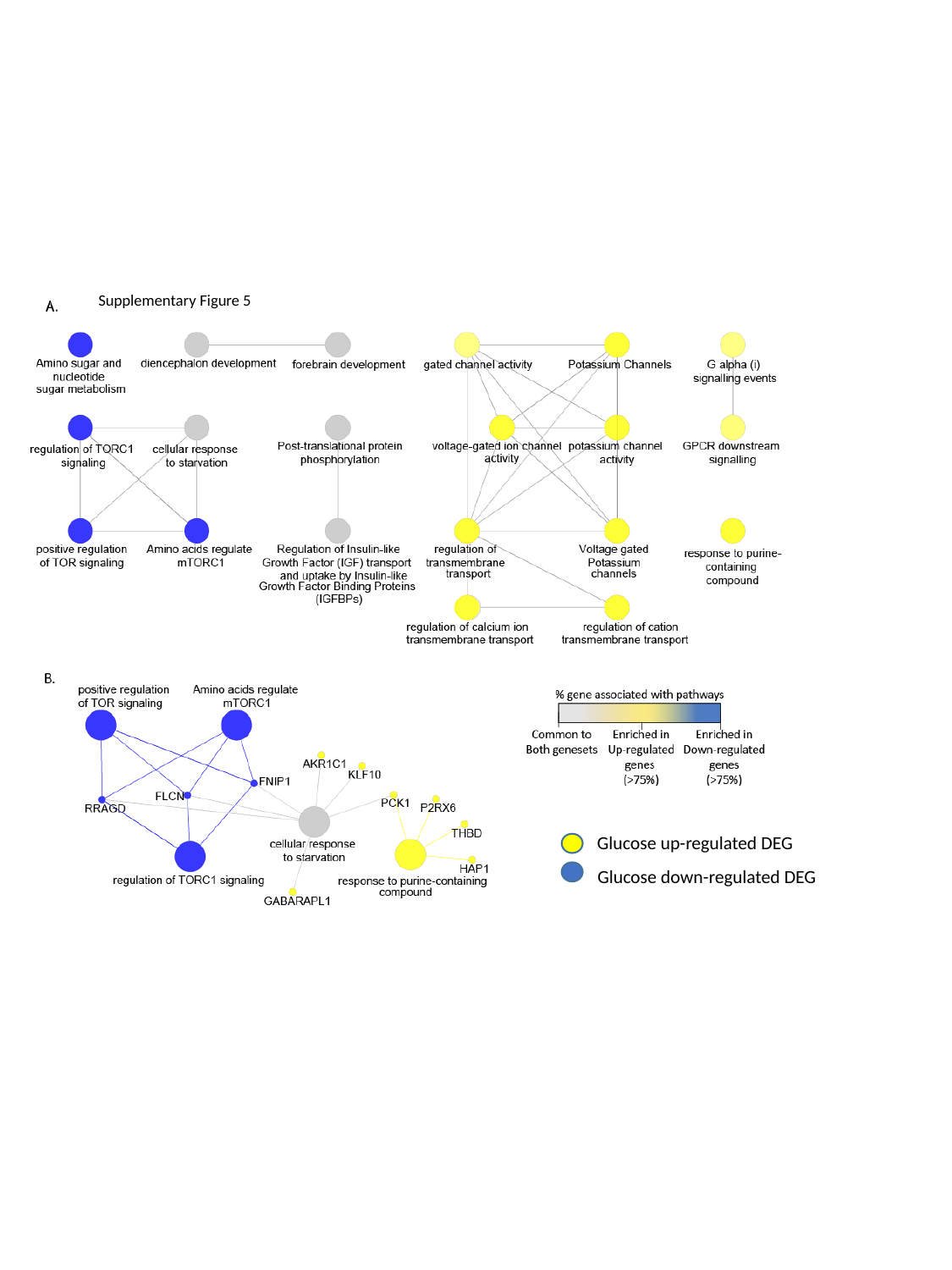

Supplementary Figure 5
Glucose up-regulated DEG
Glucose down-regulated DEG

## Slide 11
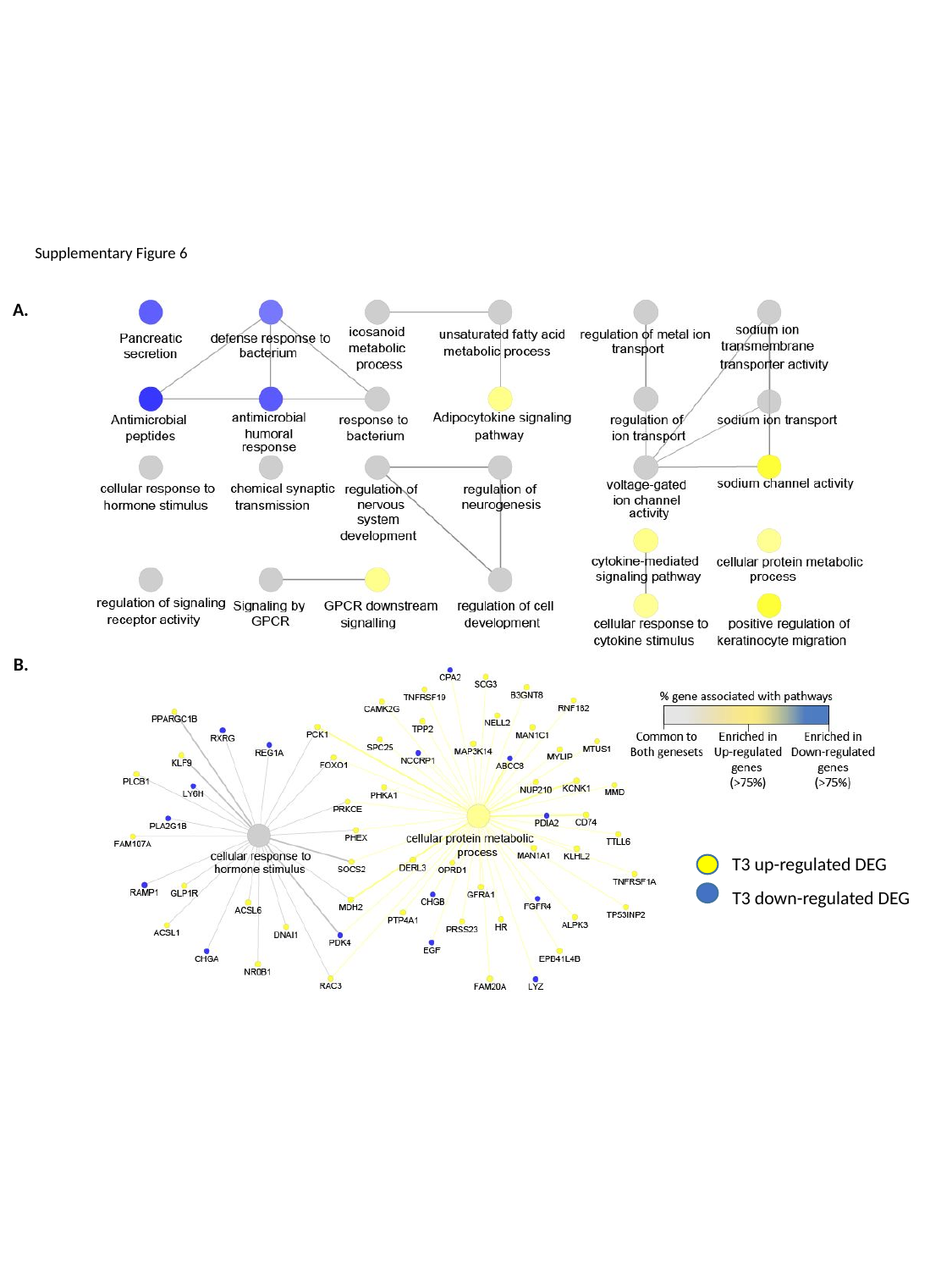

Supplementary Figure 6
A.
B.
T3 up-regulated DEG
T3 down-regulated DEG

## Slide 12
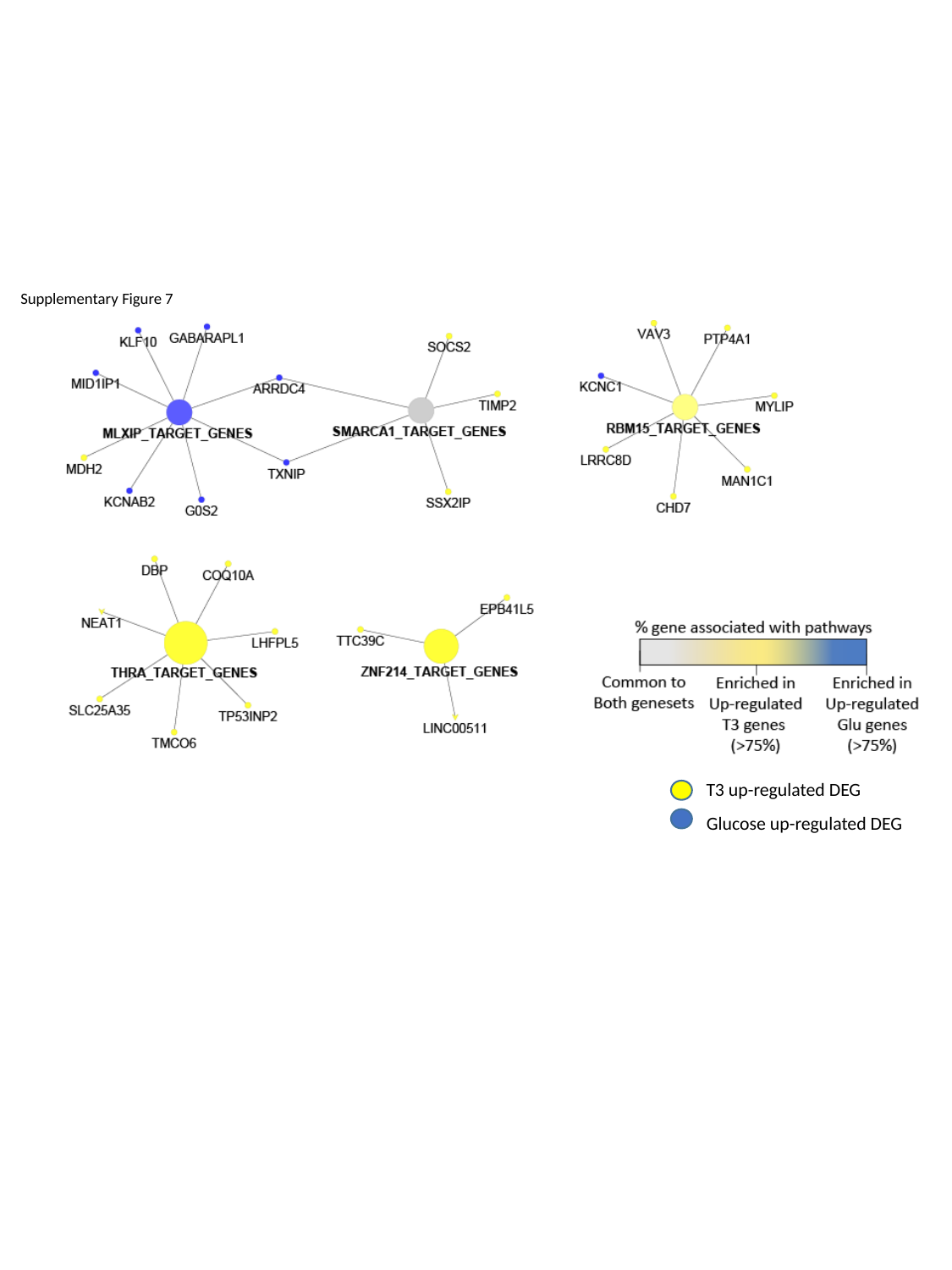

Supplementary Figure 7
T3 up-regulated DEG
Glucose up-regulated DEG

## Slide 13
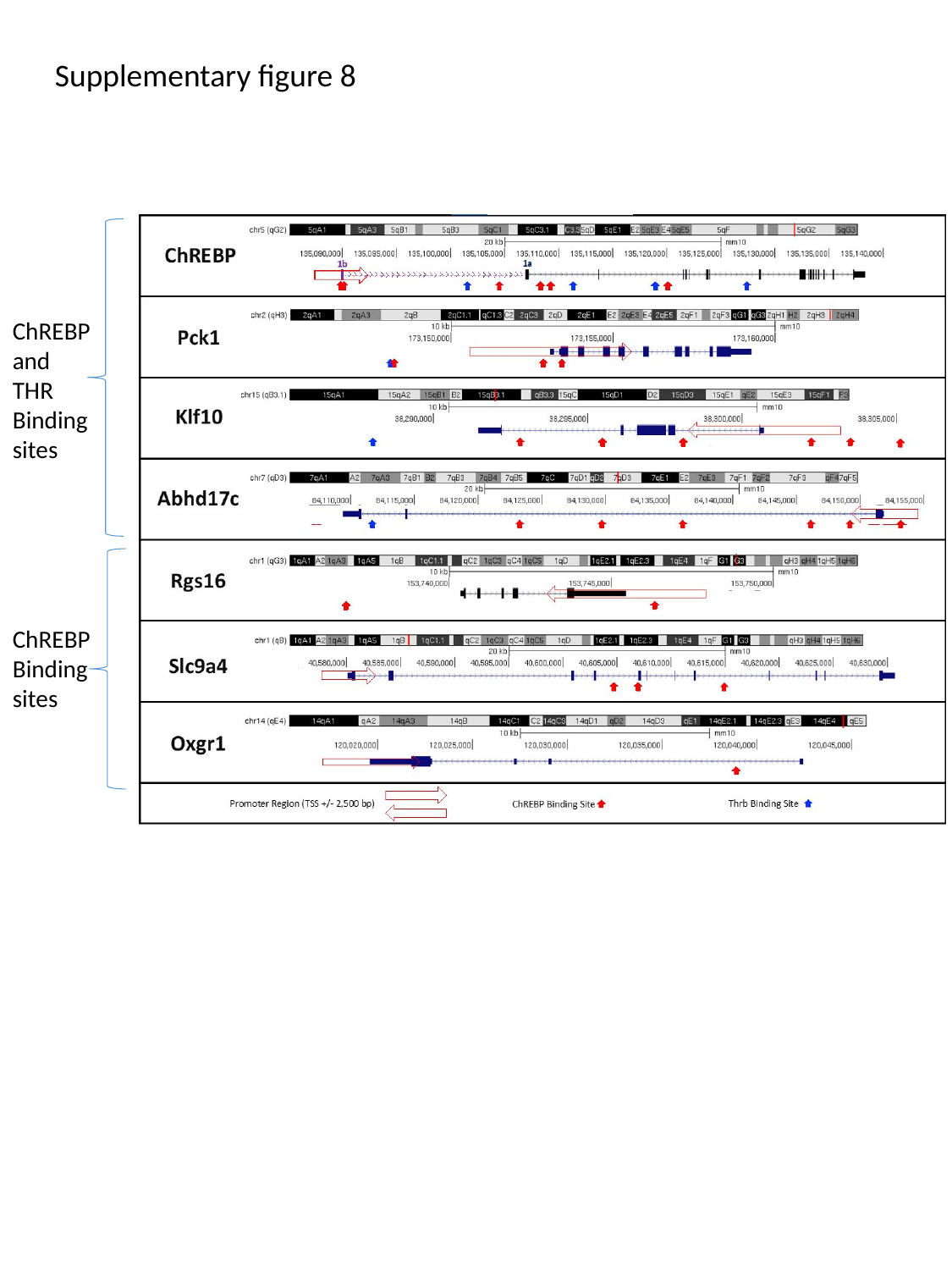

Supplementary figure 8
ChREBP and THR
Binding sites
ChREBP Binding sites

## Slide 14
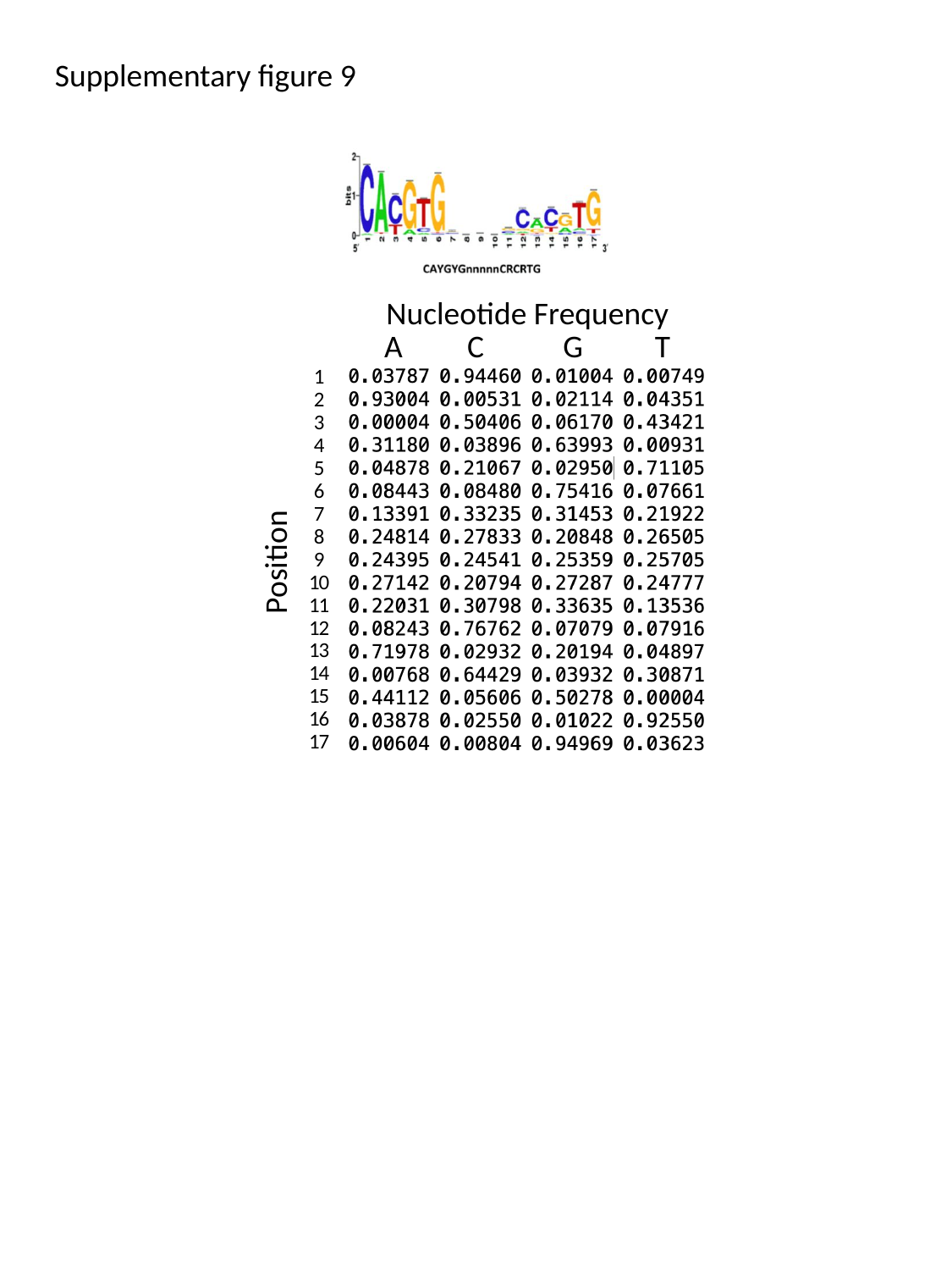

Supplementary figure 9
Nucleotide Frequency
A C G T
1
2
3
4
5
6
7
8
9
10
11
12
13
14
15
16
17
Position
