# Supplementary material for: T3 and glucose increase expression of phosphoenolpyruvate carboxykinase (PCK1) leading to increased β-cell proliferation
Source: Mol Metab. 2022 Nov 29;66:101646. doi: 10.1016/j.molmet.2022.101646 (PMC9731891; doi:10.1016/j.molmet.2022.101646)
Supplement: Multimedia component 2 [file mmc2.docx]

**Supplemental tables:**

**Supplementary table 1-** list of primers used in this study

**Supplementary table 2-** list of antibodies used in this study

**Supplementary table 3-** report the coordinates of both the carbohydrate response element binding protein and the thyroid hormone receptor beta sites for or RBRA, in the human (GRCh38/hg38) genome

**Supplementary table 4-** report the coordinates of both the carbohydrate response element binding protein and the thyroid hormone receptor beta sites for or RBRA, in the mouse (GRCm38/mm10) genome

**Supplemental Figure Legends**

**Supplemental Figure 1: Proliferation of human islets in response to T3 and glucose.** Human islets were dispersed and incubated at the indicated glucose or T3 concentrations in RPMI containing 10% resin striped serum. After 48 h, cells were fixed and immunostained for Ki67 and Insulin. Presented is the percent of ki67 (A) or Insulin (B) positive cells.. Data are the means ± SEM of at least three independent experiments. *, Statistical significance with P < 0.05 ; **, P<001; ***, P<005; ****, P<001 by one-way ANOVA.

**Supplementary Figure 2: Genes co-upregulated by both T3 and glucose.** Heatmap of relative expression levels in primary human islets of genes that are identified in this study to be upregulated by either an increase in glucose concentrations (6mM vs. 20mM glucose in the presences or absence of T3) and by an increase in T3 concentrations (0nM vs. 10nM T3, in low or high glucose concentration).

**Supplementary Figure 3: Comparing the effects of T3 or high glucose on gene expression**. A. The log fold changes (log FC) in genes found differentially expressed in T3-treated islet cultures in the presence of high glucose, or low glucose were compared. B. The logFC in genes found differentially expressed in high glucose treated cultures in the presence or absence of T3 were compared. Only genes which were found significantly differentially expressed in either condition (at BH adjp P <0.05 and nologFC cut-off) were compared.A linear trendline shows a strong correlation between the expression changes.

**Supplementary Figure 4Genes affected by high glucose and T3 treatment.** A. A volcano plot showing the log fold change (logFC) vs –log adj p value (-log adj P. Val)for genes found differentially expressed in A. high glucose or B. T3 treated islet cultures. Only a selected subset of genes is labeled. Points are colored red if log FC>0.5 and BH Adj P <0.01.

**Supplementary Figure 5: Pathway enrichment analysis of genes responsive to high glucose treatment.** A. Genes differentially expressed (either increased or decreased) upon high glucose treatment (at BH Adj P <0.05, and minimum logFC > [abs]0.25) were tested for enrichment in KEGG, Reactome of GO Biological pathways. A. Up and down-regulated genes were queried separately for pathway enrichments. Pathways found enriched with BH adj P <0.05 are shown as nodes, with the color of the nodes indicating if the pathway was more associated with up (yellow) or down (blue) regulated genes. Edges connect pathways with similar gene membership. B. A subset of the pathways in A are shown with the associated genes, colored blue or yellow, if they were down- or up- regulated with high glucose treatment.

**Supplementary Figure 6: Pathway enrichment analysis of genes responsive to T3 treatment.** A. Genes differentially expressed (either increased or decreased) upon T3 treatment (at BH Adj P <0.05, and minimum logFC > [abs]0.25) were tested for enrichment in KEGG, Reactome of GO Biological pathways. A. Up and down-regulated genes were queried separately for pathway enrichments. Pathways found enriched with BH adj P <0.05 are shown as nodes, with the color of the nodes indicating if the pathway was more associated with up (yellow) or down (blue) regulated genes.Edges connect pathways with similar gene membership. B. A subset of the pathways in A are shown with the associated genes, colored blue or yellow, if they were down- or up- regulated with T3 treatment.

**Supplementary Figure 7: Transcription factor enrichment analysis of high glucose and T3 up-regulated genes.** A. Genes differentially increased upon high glucose or T3 treatment (at BH Adj P <0.05, and minimum logFC >0.25) were tested for enrichment in MSigDB transcription factor target database GTRD (Gene Transcription Regulation Database v19.10). TF’s found significantly enriched at BH adj P>0.05 are shown with the associated genes in either yellow or blue if from the T3 or Glucose up-regulated gene sets, respectively.

**Supplementary Figure 8: Promoters of key regulatory genes for islet development contain THR and ChREBP binding sites**. ChREBP and THRB binding sites in mouse selected genes. Each panel is arranged as follows. The ideogram of the gene with its chromosomal location from the UCSC genome browser is shown. The representation displays exons (dark blue boxes) and introns (dark blue lines with arrowheads pointing to the direction of transcription). The promoter region (TSS +/- 2,500 bp) is shown as a transparent red arrow. For the ChREBP gene, the position of the additional exon 1b is marked with a purple box and the intron between exons 1b and 1a is marked with a purple line with arrowheads oriented as for the rest of the gene. Blue and red upward arrowheads identify the center of ChREBP and THRB binding sites. ChREBP binding sites have been scored with the HOMER package (see material and methods) by using the frequency matrix of supplementary figure 8, except for three sites that have been experimentally validated and are marked with asterisks near the respective arrowheads. THRB sites have been extracted from the ReMap2022 database. Supplementary table 4 provides the coordinates of both ChREBP and THRB sites displayed.

**Supplementary Figure 9:**

Sequence logo for ChORE (upper panel) and frequency matrix (lower panel), generated by the HOMER package
